# The human G protein‐coupled ATP receptor P2Y_11_ is a target for anti‐inflammatory strategies

**DOI:** 10.1111/bph.15379

**Published:** 2021-02-19

**Authors:** Georg Gruenbacher, Hubert Gander, Gabriele Dobler, Andrea Rahm, Dominik Klaver, Martin Thurnher

**Affiliations:** ^1^ Immunotherapy Unit, Department of Urology Medical University of Innsbruck Innsbruck Austria

**Keywords:** ADAM17, cyclic AMP, interleukin‐1, M2 macrophages, P2Y_11_, soluble TNF receptor, Toll‐like receptor

## Abstract

**Background and Purpose:**

The ATP receptor P2Y_11_, which couples to G_q_ and G_s_ proteins, senses cell stress and promotes cytoprotective responses. P2Y_11_ receptors are upregulated during differentiation of M2 macrophages. However, it is unclear whether and how P2Y_11_ receptors contribute to the anti‐inflammatory properties of M2 macrophages.

**Experimental Approach:**

Transcriptome and secretome profiling of ectopic P2Y_11_ receptors was used to analyse their signalling and function. Findings were validated in human monocyte‐derived M2 macrophages. The suramin analogue NF340 and P2Y_11_ receptor‐knockout cells confirmed that agonist‐mediated responses were specific to P2Y_11_ receptor stimulation.

**Key Results:**

Temporal transcriptome profiling of P2Y_11_ receptor stimulation showed a strong and tightly controlled response of IL‐1 receptors, including activation of the IL‐1 receptor target genes, *IL6* and *IL8*. Secretome profiling confirmed the presence of IL‐6 and IL‐8 proteins and additionally identified soluble tumour necrosis factor receptor 1 and 2 (sTNFR1 and sTNFR2) as targets of P2Y_11_ receptor activation. Raised levels of intracellular cAMP in M2 macrophages, after inhibition of phosphodiesterases (PDE), especially PDE4, strongly increased P2Y_11_ receptor‐induced release of sTNFR2 through ectodomain shedding mediated by TNF‐α converting enzyme (TACE/ADAM17). Both IL‐1α and IL‐1ß synergistically enhanced P2Y_11_ receptor‐ induced IL‐6 and IL‐8 secretion and release of sTNFR2. During lipopolysaccharide‐induced activation of TLR4, which shares the downstream signalling pathway with IL‐1 receptors, P2Y_11_ receptors specifically prevented secretion of TNF‐α.

**Conclusions and Implications:**

Targeting P2Y_11_ receptors activates IL‐1 receptor signalling to promote sTNFR2 release and suppress TLR4 signalling to prevent TNF‐α secretion, thus facilitating resolution of inflammation.

**Abbreviations**
CBA: cytokine bead array
DAG: diacylglycerol
GM‐CSF: granulocyte/macrophage colony‐stimulating factor
IBMX: 3‐isobutyl‐1‐methylxanthine
IKK: IkB kinase
IL‐1RAP: interleukin‐1 receptor‐associated protein
IP_3_
: inositol trisphosphate
LPS: lipopolysaccharide
MAPK: mitogen‐activated protein kinase
M‐CSF: macrophage colony‐stimulating factor
MFI: mean fluorescence intensity
MyD88: myeloid differentiation primary response 88
PBMC: peripheral blood mononuclear cell
PLC: phospholipase C
SASP: senescence‐associated secretory phenotype
sTNFR: soluble tumour necrosis factor receptor
TIR: Toll/IL‐1R
TLR: Toll‐like receptors

**What is already known**

- P2Y_11_ receptors may have pro‐ and anti‐inflammatory effects.
- P2Y_11_ receptors are upregulated during differentiation of human M2 macrophages.

**What this study adds**

- There is crosstalk between the signalling pathways of P2Y_11_ receptors, IL‐1 receptors and TLR4.
- Activation of P2Y_11_ receptors mediates suppression of TNF‐α secretion and stimulation of TNF receptor release.

**What is the clinical significance**

- P2Y_11_ receptor‐mediated control of TNF‐α is important to prevent hyperinflammation.
- Activation of P2Y_11_ receptors may be beneficial in autoimmune and infectious diseases.

## 1 INTRODUCTION

Extracellular ATP, which is a member of the damage‐associated molecular pattern (DAMP) family, is found in the inflammatory and tumour microenvironment following controlled or lytic release from stressed cells (Carta et al., 2015; Junger, 2011). The concentration of ATP in the interstitium of healthy tissues is very low (in the nanomolar range) but may accumulate to high levels, both in inflammatory lesions and in the tumour microenvironment. In vivo measurements have shown that ATP can rise dramatically at sites of inflammation (low to high micromolar range), leading to the activation of ATP‐responsive P2 receptors (Di Virgilio et al., 2020).

The P2Y_11_ receptor is an unconventional member of the P2Y family of G protein‐coupled receptors (GPCRs) (Jacobson et al., 2020). According to the current view, P2Y_11_ receptors sense extracellular ATP and translate this prototypical danger signal into cytoprotective and immunomodulatory responses (Kennedy, 2017; Vitiello et al., 2012). Expression of P2Y_11_ receptors has been detected in immune (Dreisig et al., 2018; Marteau et al., 2005) and non‐immune cells (Benoist et al., 2019; Lefort et al., 2018), including cancer cells (Khalid et al., 2017). Among immune cells, P2Y_11_ receptors may preferentially be expressed in cells of myeloid origin such as dendritic cells (Schnurr et al., 2003; Wilkin et al., 2001). However, recent work has demonstrated that P2Y_11_ receptor expression substantially increases during M2 macrophage differentiation, both at the mRNA (Layhadi & Fountain, 2019) and at the protein level (Gruenbacher et al., 2019). Alternatively activated M2 macrophages are required to regulate or completely switch off inflammation (Martinez et al., 2008). Failure to control the response of pro‐inflammatory M1 macrophages is known to contribute to inflammatory disease including hyperinflammation associated with coronavirus disease 2019 (COVID‐19) (Mehta et al., 2020; Merad & Martin, 2020; Schulert & Grom, 2015). In particular, TNF receptor signalling requires tight control as sustained activation of the pathway may lead to inflammatory diseases (Chen & Goeddel, 2002; Verstrepen et al., 2008) and contribute to severe respiratory failure (SRF) in COVID‐19 patients (Giamarellos‐Bourboulis et al., 2020; Huang et al., 2020; Netea et al., 2020).

While expression of P2Y_11_ receptors increased during M2 macrophage differentiation (Gruenbacher et al., 2019), it remained unclear whether and how these receptors may contribute to the anti‐inflammatory properties of M2 macrophages. P2Y_11_ receptors cannot be studied in vivo as no murine receptor has yet been cloned, suggesting that rodents do not have a functional P2Y_11_ receptor (Dreisig & Kornum, 2016). In the present work, we have therefore studied ectopic P2Y_11_ receptors in a recombinant human cell system as well as native P2Y_11_ receptors in human monocyte‐derived M2 macrophages using transcriptomic and proteomic approaches. We provide strong evidence for P2Y_11_ receptor crosstalk with the IL‐1 receptor/TLR4 signalling pathway. In M2 macrophages, P2Y_11_ receptors cooperated with IL‐1 receptors to promote the release of sTNFR2 and blocked TLR4 to decrease TNF‐α secretion. Our work thus establishes the P2Y_11_ receptors as a potent sentinel system in the surveillance of TNF‐α driven inflammation and as a target for anti‐inflammatory strategies.

## 2 METHODS

### 2.1 Ectopic P2Y_11_ receptor expression

To study ectopic P2Y_11_ receptors, we took advantage of a commercial recombinant cell line intended for drug discovery (ES‐293‐A from PerkinElmer). This stable recombinant cell line, which is naturally devoid of functional P2 receptors, has been transfected with human P2Y_11_ receptors. The cDNA coding for the human P2Y_11_ receptor (AF030335.1) (Communi et al., 1997) has been cloned as an EcoRI‐XbaI fragment into the bicistronic pEFIN3 vector for constitutive expression from an EF1alpha promoter. The neomycin gene (conferring G418 resistance) is driven by an IRES sequence placed immediately after the P2Y_11_ receptor‐coding cDNA (Perkin Elmer). By using CRISPR/Cas9‐mediated gene knockout, we previously generated an appropriate control cell line, which lacks P2Y_11_ receptors (P2RY11‐KO) (Gruenbacher et al., 2019). The cell lines were grown at 37°C in a humidified 5% CO_2_ atmosphere in Dulbecco's modified Eagle's medium (DMEM) supplemented with 10% (v/v) FBS, 1 mM sodium pyruvate, 2 mM l‐alanyl‐l‐glutamine (Glutamax), 100 units·ml^−1^ of penicillin, and 100 μg·ml^−1^ of streptomycin. G418 (geneticin) was used at 400 μg·ml^−1^ to select for stable transfectants as well as knockouts. Both the P2Y_11_ receptor‐expressing cell line and the knockout control cell line were regularly sorted on a BD FACSAria at the local FACS core facility to select for presence (P2RY11) or absence of P2Y_11_ (P2RY11‐KO), respectively.

### 2.2 Transcriptome analysis

Total RNA was isolated from six‐well plate derived P2RY11 and P2RY11‐KO cell pellets using the RNeasy Plus Micro Kit (Qiagen) according to the manufacturer's instructions. Total RNA samples were quality checked on an Agilent Bioanalyzer 2100 (Foster City, CA) with an RNA 6000 Nano LabChip and samples with RNA integrity factors above 8.0 were used for further analysis; 50 ng total RNA were used for hybridisation reaction with the nCounter PanCancer Human Pathways Panel Kit and with the nCounter PanCancer Immune Profiling Panel Kit according to supplier's instructions (NanoString Technologies, Seattle, WA USA; Biomedica, Vienna, Austria). Samples were processed at the Core Facility Molecular Biology at the Centre of Medical Research at the Medical University of Graz. The automated NanoString platform is based on fluorescent barcodes and digital readout allowing for the non‐amplified measurement of 730 protein‐coding mRNA sequences within one sample. The NanoString platform has been shown to be comparable with other technologies, with considerable sensitivity, reproducibility, and technical robustness (https://www.nanostring.com/scientific-content/publications).

Raw data pre‐processing and normalisation was performed using nSolver 2.5 Software (NanoString Technologies, Seattle, WA USA) according to standard procedures. After this, gene counts were normalised to the geometric mean of the 40 reference genes. Normalised data were uploaded to Partek Genomic Suite Software v6.6 (Partek Inc., St. Louis, MO). For statistical analysis, one‐way ANOVA was calculated and genes with *P* <.05 and fold change of at least 1.5 were considered as differentially regulated.

### 2.3 Secretome analysis

The Human Cytokine Antibody Array Kit G2000 was used to detect 174 cytokines. The G2000 comprises the AAH‐CYT‐G6, AAH‐CYT‐G7, and AAH‐CYT‐G8 arrays, which have a glass side layout and utilise a fluorescent readout. The specific capture antibodies are immobilised to glass slides. After a blocking step, samples were incubated with the arrays. Non‐specific proteins were then washed off, and the arrays were incubated with detection antibodies. After an additional wash step, glass‐based arrays were subjected to laser scanning (Innopsys, InnoScan). A complete list of the analytes can be found in a supplementary file. After subtracting background signals and normalisation to internal positive and negative controls, comparison of signal intensities for antigen‐specific antibody spots between and among array images was used to determine relative differences in expression levels of each analyte between samples: untreated, P2Y_11_ receptor agonist (ATPγS), ATPγS plus P2Y_11_ receptor antagonist (NF340), NF340 alone.

To validate candidates emerging from the secretome analysis, we performed cytokine bead arrays (CBAs) from BD. Cytokine levels in cell culture supernatants were assessed 24 h after cell stimulation using human CBA Flex Sets for IL‐1α, IL‐1ß, IL‐6, IL‐8, IL‐10, TNF‐α, sTNFR1, sTNFR2, and CCL2 (MCP‐1). Samples were analysed with a FACSCanto II system and FCAP Array 1.0.1 software from BD.

### 2.4 siRNA‐mediated IL1R1 gene knockdown

IL1R1 gene knockdown in P2RY11 cells was achieved by RNA interference using the ON‐TARGETplus SMARTpool (Dharmacon, Horizon Discovery, L‐005188‐00‐0005) containing four different IL1R1 targeting siRNAs (GAACACAAAGGCACUAUAA, GCAAAUAGCCAUGUAUAAU, CAUCACAGUGCUUAAUAUA, GGACUUGUGUGCCCUUAUA). The ON‐TARGETplus nontargeting control pool (Dharmacon, D‐001810‐10‐05) containing four different siRNAs with no significant homology to any human mRNA (UGGUUUACAUGUCGACUAA, UGGUUUACAUGUUGUGUGA, UGGUUUACAUGUUUUCUGA, UGGUUUACAUGUUUUCCUA) served as negative control. The transfection of P2RY11 cells with respective siRNAs was carried out using Lipofectamine RNAiMAX transfection reagent (Invitrogen) according to the manufacturer's instructions. Briefly, P2YR11 cells were seeded at 3–4 × 10^4^ cells per well in a 48‐well plate (Costar) in full growth medium (DMEM supplemented with 10% (v/v) FBS, 1 mM sodium pyruvate, 2 mM l‐alanyl‐l‐glutamine (Glutamax), 100 units·ml^−1^ of penicillin, 100 μg·ml^−1^ of streptomycin, and 400 μg·ml^−1^ of G418) 24 h prior to transfection. The next day, medium was replaced by 200 μl transfection medium (DMEM supplemented with 10% [v/v] FBS, 1 mM sodium pyruvate, 2 mM l‐alanyl‐l‐glutamine [Glutamax] without antibiotics) when cells were approximately 40%–50% confluent. Next, siRNA‐lipid complexes were generated in serum‐free OptiMEM (Invitrogen) by addition of diluted siRNA with a final concentration of 50 nM to diluted Lipofectamine RNAiMAX reagent (0.8 μl per transfection) in a 1:1 ratio. After 15–20 min incubation, cells were forward transfected in triplicates by dropwise addition of 40 μl siRNA‐lipid complexes to each well, and culture plates were rocked gently to ensure equal distribution of the complexes. Transfected cells were incubated at 37°C for 24 h; after which, the transfection medium was replaced by 400 μl full growth medium in order to avoid cytotoxicity of the transfection reagent. Moreover, transfection efficiency was controlled after 24 h using siGLO RISC‐Free Transfection Indicator (Dharmacon, D‐001630‐01‐05), which was >85%. In addition, the level of knockdown was assessed at the protein level 72 h post transfection via quantification of IL‐1R1 surface expression by flow cytometry.

### 2.5 Monocyte isolation and macrophage differentiation

Buffy coats representing residual blood apheresis products dedicated to disposal served as starting material for the isolation of human peripheral blood mononuclear cells (PBMCs). Buffy coats from randomly selected regular healthy donors were provided in an anonymous way by the Central Institute for Blood Transfusion (Innsbruck, Austria) after written informed consent. Therefore, we have no access to data on age or sex of our blood donors. Inclusion of healthy donors in the present study was approved by the local Institutional Review Board (ethical committee number: 1087/2018).

PBMCs were isolated from buffy coats by density gradient centrifugation (Lymphoprep). Monocytes were subsequently isolated using CD14 microbeads and LS columns from Miltenyi Biotec (purity >95%). M2 macrophages were differentiated from freshly isolated monocytes for 5 to 6 days in RPMI1640 containing 10% FBS in the presence of macrophage colony‐stimulating factor (M‐CSF) (50 ng·ml^−1^) with addition of fresh M‐CSF containing medium on Day 2 and Day 5.

### 2.6 M2 macrophage stimulation

Fully differentiated M2 macrophages were harvested and washed, and 5 × 10^4^ cells were stimulated in 100 μl of RPMI1640 containing 5% FBS on Day 6 with P2Y_11_ receptor agonists (ATPγS, NF546; 1 to 10 μM) or LPS (10 pg to 10 ng·ml^−1^) in the presence or absence of antagonists/inhibitors for 24 h. Supernatants were harvested and cryopreserved at −80°C.

### 2.7 Flow cytometry

The antibody‐based procedures used in this study comply with the recommendations made by the *British Journal of Pharmacology*. Cell surface antigens were stained with fluorochrome‐conjugated monoclonal (mouse) or polyclonal (rabbit, goat) antibodies. The respective isotype control was tested in parallel using the same concentration. The following antibodies were used for macrophage phenotyping: rabbit polyclonal IgG anti‐human P2Y_11_ receptor (Bioss/THP, bs‐12071R‐A488, RRID:AB_2857964), rabbit polyclonal anti‐human P2X1 receptor (Bioss/THP, bs‐12107R‐A647, custom‐conjugation), mouse monoclonal IgG2b anti‐human CD14 (BD Bioscience, clone MφP9, 345,787‐APC, RRID:AB_400509), mouse monoclonal IgG1k anti‐human CD163 (BD Bioscience, clone GHI/61, 556,018‐PE, RRID:AB_2033943), mouse monoclonal IgG2a anti‐human CD284 (TLR4) (BioLegend, clone HTA125, 312,816‐APC, RRID:AB_2562487), goat polyclonal IgG anti‐human IL‐1R1 (R&D Systems/Bio‐techne, FAB269P‐PE, RRID:AB_2124912); mouse monoclonal IgG1 anti‐human P2Y_11_ receptor (R&D, clone # 505214‐A647, RRID:AB_2857965) was used to detect human P2Y_11_ receptor on glioma cells after gentle cell detachment using Accutase (Sigma‐Aldrich).

The harvested cells were washed (0.3×*g* for 5 min) and then stained on ice for 30 min in the dark in PBS containing 0.5% FCS and 50 μg·ml^−1^ human IgG (Octapharma) to block Fc γ receptors. Fixable viability dye eFluor 780 (Thermo Fisher Scientific) was used to label dead cells. For all samples, acquisition and analysis was performed on a FACSCanto II flow cytometer and FACS Diva 6.1.2 as well as FlowJo V7.2.5 software (RRID:SCR_008520) (BD Biosciences) by applying dead cell and doublet discrimination.

### 2.8 Data and statistical analysis

The data and statistical analysis comply with the recommendations on experimental design and analysis in pharmacology (Curtis et al., 2018). For studies of M2 macrophages, a total of 12 blood donors were included. All the studies were designed to generate groups of equal sample size, using randomisation and blinded analysis. Data are presented as mean ± SEM of five to eight independent assays with at least three replicates. Data are presented as bar charts because scatter plot or before–after charts did not reveal unusual or interesting aspects of the data not obvious from the bar chart. Statistical analyses were performed using GraphPad Prism software (version 8.4.1, RRID:SCR_002798). ANOVA was used for comparing means of more than two groups. Post‐hoc tests were conducted only if *F* was significant and there was no variance heterogeneity. A value of *P* <.05 was accepted as showing significant differences in all tests.

### 2.9 Materials

ATPγS (Sigma Aldrich, St. Louis, MO, USA) and NF546 (Tocris, Bristol, UK) were used as P2Y_11_ receptor agonists (10 to 30 μM). NF340 (Santa Cruz, Dallas, TX, USA) served as P2Y_11_ receptor antagonist (10 μM) (Gruenbacher et al., 2019). Additional reagents used in this study are as follows: P2X1 receptor antagonist NF449 (10 μM) (Tocris), adenylyl cyclase inhibitor SQ22536 (5 to 20 μM) (Tocris), Gq/11 inhibitor YM‐254890 (0.1 to 0.5 μM) (Adipogen Life Sciences, Liestal, Switzerland), recombinant IL‐1α and IL‐1ß (50 to 1,000 pg·ml^−1^) (R&D, Bio‐Techne), recombinant IL‐1 receptor antagonist (IRAP; 0.05 to 0.2 μg·ml^−1^) (R&D, Bio‐techne); nonselective phosphodiesterase inhibitor 3‐isobutyl‐1‐methylxanthine (IBMX) (20 to 200 μM) (Sigma‐Aldrich); PDE4‐selective inhibitor rolipram (2 to 10 μM) (Sigma‐Aldrich), the TACE/ADAM17 inhibitors TAPI‐1 and TAPI‐2 (10 to 50 μM) (Tocris); lipopolysaccharide (LPS) from Salmonella abortus equi (10 pg·ml−1 to 10 ng·ml^−1^) (Sigma‐Aldrich) was used to activate M2 macrophages via TLR4.

### 2.10 Nomenclature of targets and ligands

Key protein targets and ligands in this article are hyperlinked to corresponding entries in the IUPHAR/BPS Guide to PHARMACOLOGY (http://www.guidetopharmacology.org) and are permanently archived in the Concise Guide to PHARMACOLOGY 2019/20 (Alexander, Christopoulos, et al., 2019; Alexander et al., 2019a, 2019b).

## 3 RESULTS

### 3.1 Temporal transcriptional network analysis of P2Y_11_ receptor activation revealed the signature of strong but transient IL‐1 receptor/TLR signalling

**FIGURE 1:**
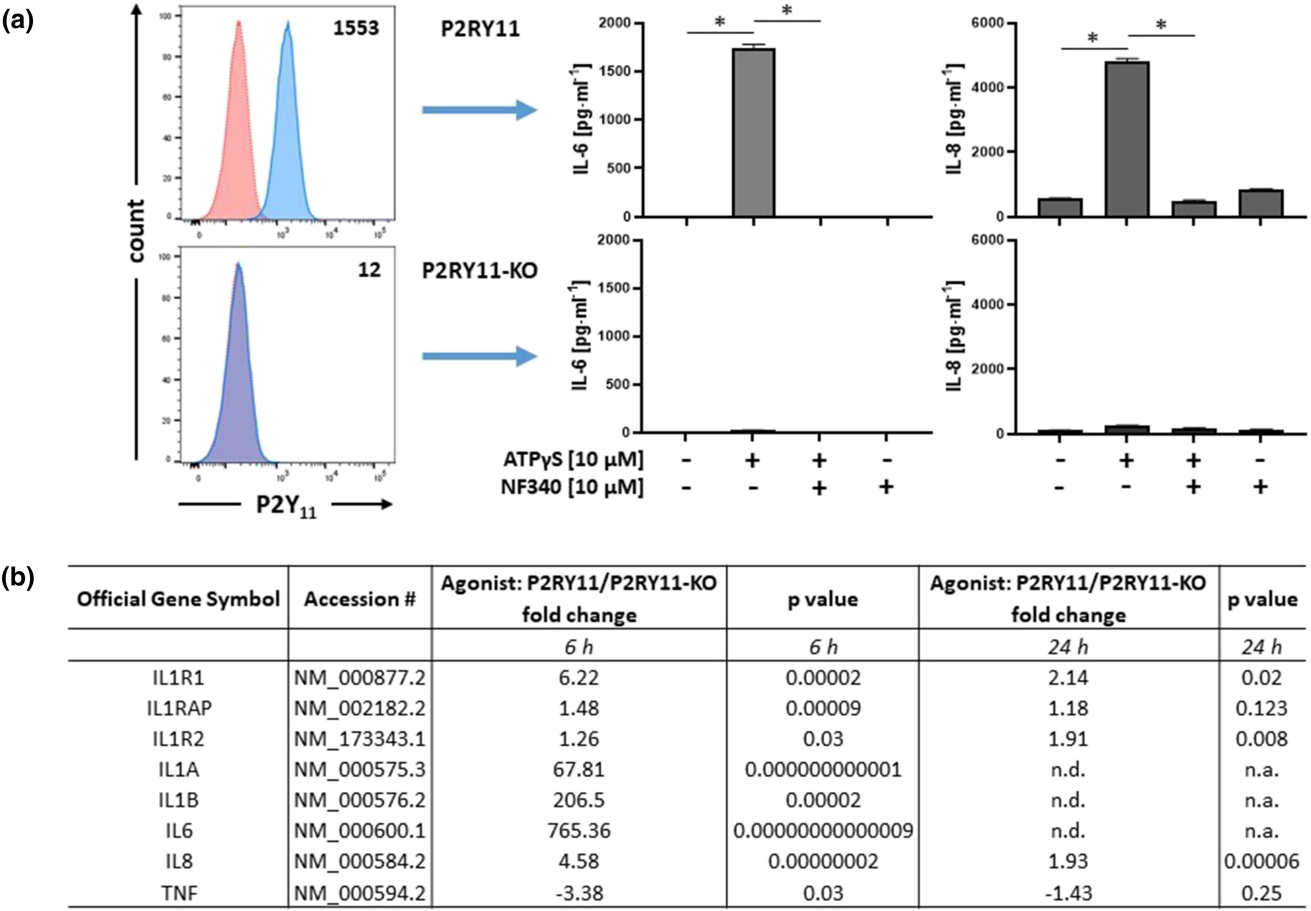
Ectopic P2Y_11_ receptor signalling activates genes related to IL‐1 receptor /TLR4 pathways. (a) Surface expression of P2Y_11_ receptors was measured by flow cytometry in the recombinant cell line (P2YR11) and in the P2RY11 knockout control (P2RY11‐KO). Numbers are mean fluorescence intensities (MFIs) of P2Y_11_ staining after subtraction of isotype control MFIs. P2RY11‐transfected cells and P2RY11 knockout cells (P2RY11‐KO) were treated for 24 h with ATPγS at 10 μM. IL‐6 and IL‐8 were measured in cell culture supernatants. The antagonist NF340 was used at 10 μM to confirm that agonist‐mediated responses were specific to stimulation of P2Y_11_ receptors (*n* = 5). *P < .05 significantly different as indicated; one‐way ANOVA. (b) Transcriptional reprogramming in response to P2Y_11_ receptor activation was examined using NanoString analysis. P2RY11‐transfected cells and P2RY11‐KO cells were cultured for 6 h and 24 h in the presence of the P2Y_11_ receptor agonist ATPγS at 10 μM (three replicates for each condition). Fold change indicates the averaged ratio of expression in agonist‐treated P2RY11 cells to that in agonist‐treated P2RY11‐KO cells (agonist: P2RY11/P2RY11‐KO). *P* values shown were derived from one‐way ANOVA. n.d., not detectable; n.a., not applicable

To obtain clues for downstream mechanisms activated by P2Y_11_ receptor signalling (Figure S1), we combined ectopic receptor expression (Figure 1a) with transcriptional profiling of P2Y_11_ receptor activation (Figure 1b), which has not been done before. We performed NanoString analyses 6 and 24 h after P2Y_11_ stimulation using the Immune Profiling Panel. The transcriptional response to P2Y_11_ receptor activation revealed a strong signature of IL‐1 receptor/TLR pathway activation (Dunne & O'Neill, 2003) (Figure 1b), including upregulation of IL6 and IL8, which are canonical target genes of IL‐1 receptor R/TLR signalling (Weber et al., 2010). The gene encoding the adaptor protein MyD88, which bridges the receptor complex to downstream signalling events (Figure S1), was also upregulated but remained below the threshold of 1.5 (1.21‐fold, *P* = .003). P2Y_11_ receptor‐driven IL‐1 receptor/TLR signalling appeared to be tightly controlled, as expression of IL1A, IL1B, and IL6 became undetectable at 24 h (Figure 1b). IL1R2 was upregulated at 24 h, suggesting that this prototypical decoy receptor is involved in the termination of IL‐1 receptor signalling.

To verify the transcriptional signature of IL‐1 receptor/TLR signalling, we measured cytokine levels. ATPγS at 10 μM induced IL‐6 and IL‐8 secretion in cells expressing P2Y_11_ receptors (P2RY11 cells) but not in knockout cells (P2RY11‐KO cells) (Figure 1a). Moreover, ATPγS induced IL‐6 and IL‐8 secretion in P2RY11 cells in a dose‐dependent manner (2 μM to 50 μM) (Figure S2A), and NF340 (10 μM), which is currently the most useful antagonist at the P2Y_11_ receptor (Dreisig & Kornum, 2016; Kennedy, 2017; Meis et al., 2010), inhibited ATPγS‐induced IL‐6 and IL‐8 secretion (Figures 1a and S2A). Inhibition of agonist‐induced responses by NF340 in P2RY11 cells and the lack of response to agonist in P2RY11‐KO cells collectively indicated that ATPγS‐induced IL‐6 and IL‐8 secretion were specific to stimulation of P2Y_11_ receptors.

**FIGURE 2:**
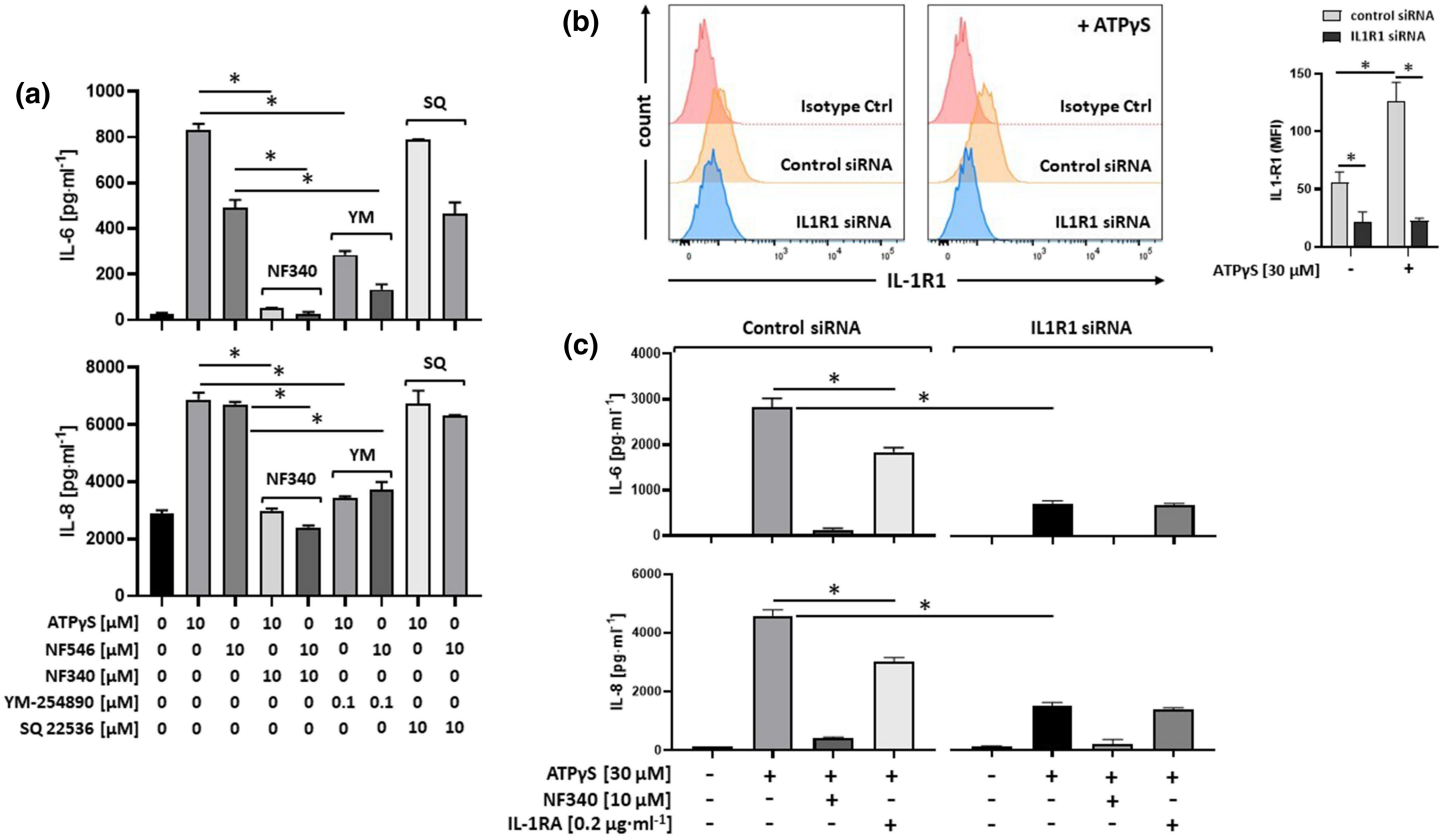
P2Y_11_ receptors preferentially couple to G_q_ and drive cytokine production in an IL‐1 receptor‐dependent manner. (a) IL‐6 and IL‐8, which are targets of IL‐1 receptor signalling, were measured in cell culture supernatants 24 h after stimulation with P2Y_11_ receptor agonists (ATPγS and NF546). The antagonist NF340 was used to confirm that agonist‐mediated responses were specific to stimulation of P2Y_11_ receptors and inhibitors were used to examine G protein coupling of P2Y_11_ receptors (G_q_: YM‐254890; G_s_: indirectly via adenylyl cyclase: SQ 22536). *P ≤ .05, significantly different as indicated; one‐way ANOVA. (b) P2RY11 cells were transfected with either control siRNA or IL1R1 siRNA for 72 h, with or without subsequent P2Y_11_ stimulation (ATPγS at 30 μM, 24 h). The level of knockdown was quantified by measuring IL‐1R1 protein surface expression by flow cytometry (left panel). Isotype control MFIs were subtracted from each staining and mean values of MFIs were calculated (*n* = 5) (right panel). *P ≤ .05, significantly different as indicated; one‐way ANOVA. (c) P2RY11 cells were transfected with either control siRNA or IL1R1 siRNA for 72 h and then treated for 24 h with P2Y_11_ agonist (ATPγS) in the presence or absence of either P2Y_11_ antagonist NF340 or recombinant human IL‐1 receptor antagonist (IL‐1RA). IL‐6 and IL‐8 were measured in cell culture supernatants. Data are means ± SEM from five independent cell populations. *P ≤ .05, significantly different as indicated; one‐way ANOVA

NF546 is known to activate P2Y_11_ receptor, although it belongs to a structural class of antagonists (suramin analogues) (Jacobson et al., 2020; Meis et al., 2010). NF546 at 10 μM also induced IL‐6 and IL‐8 production in an NF340‐sensitive manner (Figure 2a). The P2Y_11_ receptor couples to both phospholipase C (PLC) and adenylyl cyclase (AC) via Gα_q_ and Gα_s_ proteins, respectively (Communi et al., 1997) (Figure S2B). The specific G_q/11_ inhibitor YM254890 (0.1 μM) (Takasaki et al., 2004) potently inhibited ATPγS/NF546‐induced IL‐6 and IL‐8 production (Figure 2a). In contrast, the AC inhibitor SQ22536 (10 μM) had little or no effect, indicating that G_q_ coupling of P2Y_11_ receptors is required for the induction of IL‐6 and IL‐8 expression. In accordance with transcriptional downregulation of TNF (Figure 1b), TNF‐α protein could not be detected, either in control cultures or after stimulation of P2Y_11_ receptors.

### 3.2 P2Y_11_ receptors stimulate IL‐6 and IL‐8 secretion in an IL‐1 dependent manner

Unexpectedly, we could not detect IL‐1α and IL‐1ß in response to P2Y_11_ receptor stimulation. This may be due to the potency of these cytokines, that is, the ability to induce effects at low concentrations, and the fact that the low amounts of IL‐1 are consumed during the response. However, it may also relate to their dual functionality, which refers to the broadly recognised extracellular actions of IL‐1 mediated by IL‐1 receptors on the one hand and, on the other, to the more enigmatic intracellular role of IL‐1 in transcriptional regulation (Luheshi et al., 2009).

To address the role of IL‐1α and IL‐1ß in P2Y_11_ receptor‐induced IL‐6 and IL‐8 production, we used the IL‐1 receptor antagonist (IRAP) to prevent binding of IL‐1α and IL‐1ß to IL‐1R1 (Dunne & O'Neill, 2003). The antagonist, IRAP (0.05 to 0.2 μg·ml^−1^) partly inhibited P2Y_11_ receptor‐driven IL‐6 and IL‐8 secretion (Figure S2C), suggesting extracellular and intracellular actions of IL‐1.

To clarify the role of IL‐1R1 in P2Y_11_ signalling, we performed IL1R1 gene knockdown in P2RY11 cells using RNA interference. siRNA‐mediated knockdown of IL1R1 resulted in reduced surface expression of IL‐1R1 (Figure 2b) and in markedly reduced IL‐6 and IL‐8 production, in response to stimulation of P2Y_11_ receptors (Figure 2c). Importantly, upon knockdown of IL1R1, IRAP no longer had an inhibitory effect. In these experiments, we also observed upregulation of IL‐1R1 expression in response to P2Y_11_ receptor stimulation (Figure 2b), which was in accordance with our transcriptional network data (Figure 1b).

### 3.3 P2Y_11_ receptors support IL‐1 receptor signalling

**FIGURE 3:**
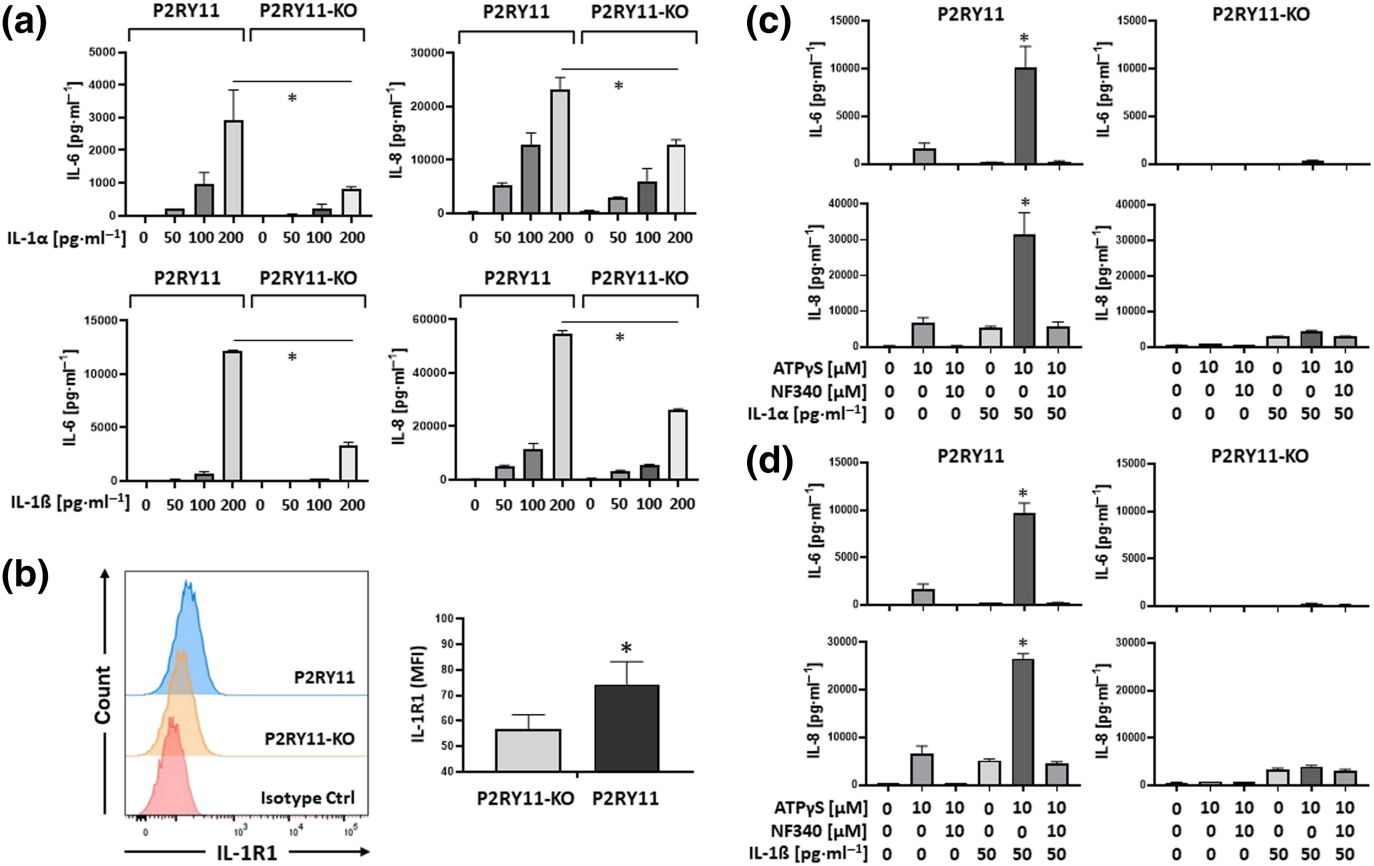
Ectopic P2Y_11_ enhances IL‐1R signalling. (a) P2RY11‐transfected cells and P2RY11 knockout cells (P2RY11‐KO) were treated for 24 h with increasing doses of IL‐1α or IL‐1ß. IL‐6 and IL‐8 were measured in cell culture supernatants. *P ≤ .05; (b) flow cytometric detection of IL‐1R1 on the surface of P2RY11‐transfected cells and P2RY11 knockout cells (P2RY11‐KO) using fluorochrome‐conjugated IL‐1R1 specific antibody and an appropriate isotype control (left panel). Quantification of eight independent stainings at different time points is shown in the right panel. Isotype control MFIs were subtracted from each staining and mean values of MFIs were calculated (*n* = 8). *P ≤ .05; (c,d) P2RY11‐transfected cells and P2RY11 knockout cells (P2RY11‐KO) were treated for 24 h with low doses of IL‐1α (c) or IL‐1ß (d) either alone or in combination with P2Y_11_ agonist. The antagonist NF340 was used to confirm that agonist‐mediated responses were specific to P2Y_11_ stimulation. IL‐6 and IL‐8 were measured in cell culture supernatants. Data shown are means ± SEM from five independent cell populations. *P ≤ .05, significantly different as indicated; one‐way ANOVA

Next, we examined effects of exogenous IL‐1. Both IL‐1α and IL‐1ß alone induced IL‐6 and IL‐8 production in a dose‐dependent manner and 50 pg·ml^−1^ of IL‐1 was sufficient to induce cytokine production (Figure 3a). Intriguingly, the P2Y_11_ receptor‐expressing cell line (P2RY11) always produced more cytokines in response to IL‐1 receptor stimulation compared to the knockout control cell line (P2RY11‐KO) (Figure 3a). Consistent with this observation, our flow cytometric analysis revealed higher levels of IL‐1R1 on the surface of P2RY11 cells than on P2RY11‐KO cells (Figure 3b).

### 3.4 P2Y_11_ receptor signalling enhances IL‐1 effects in a synergistic manner

To test the possibility that P2Y_11_ receptor signalling potentiated the effects of IL‐1, we performed concurrent stimulation of P2Y_11_ and IL‐1 receptors in P2RY11 and P2RY11‐KO cells. To reveal P2Y_11_‐mediated stimulatory effects, we kept the IL‐1 concentration low (50 pg·ml^−1^). In fact, ATPγS‐mediated P2Y_11_ activation strongly enhanced IL‐6 and IL‐8 production induced by either IL‐1α (Figure 3c) or IL‐1ß (Figure 3d). Importantly, the synergistic enhancement of IL‐1 responses by ATPγS could be inhibited with NF340 and was not observed in P2RY11‐KO cells (Figure 3c,d).

**FIGURE 4:**
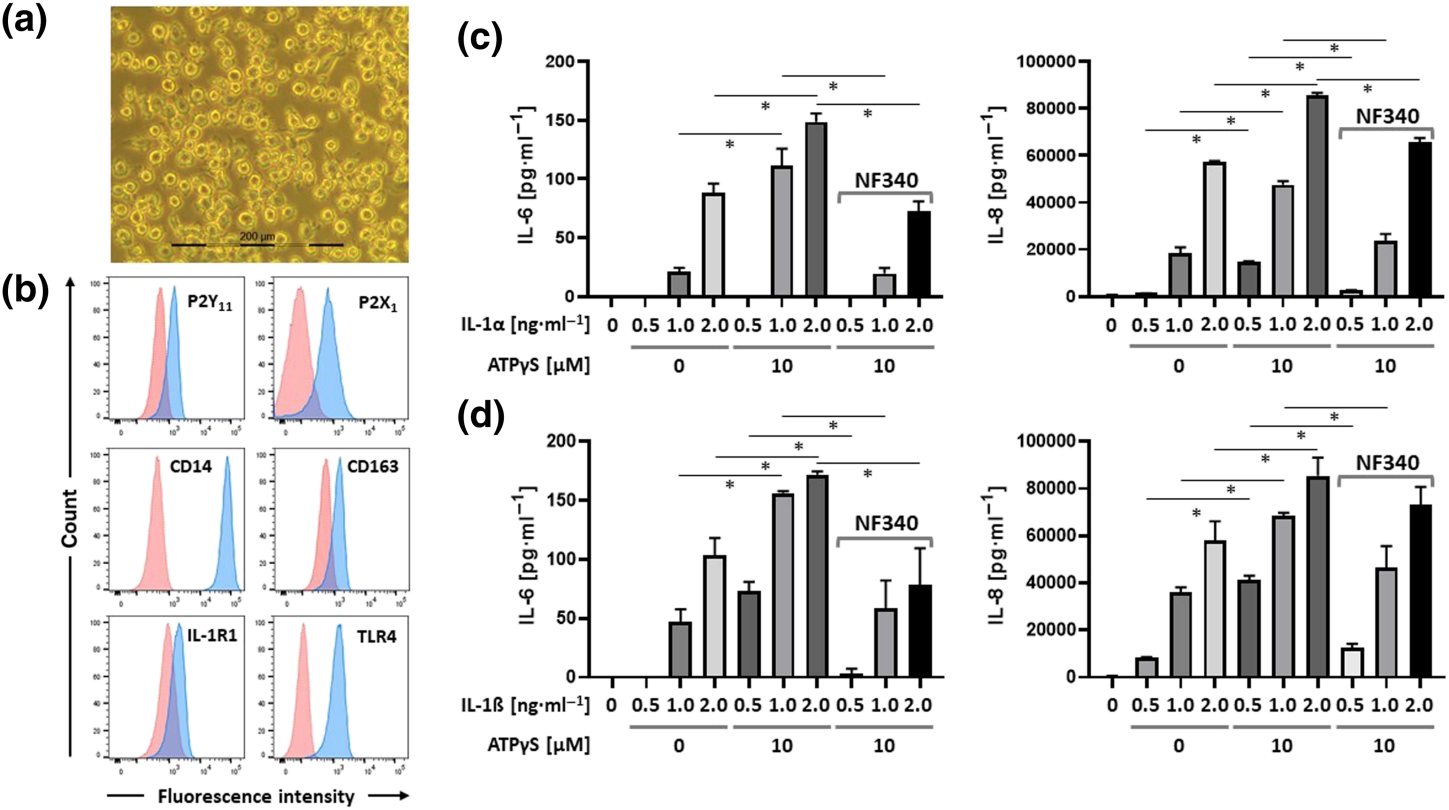
Activation of native P2Y_11_ receptors enhances IL‐1 receptor signalling. (a) Phase contrast microscopy of Day‐6 M2 macrophages (magnification: 40×). Primary human monocytes were obtained from peripheral blood mononuclear cells (PBMCs) by magnetic‐activated cell sorting (MACS) using CD14 microbeads. M2 macrophages were differentiated by culturing the isolated monocytes with M‐CSF (50 ng·ml^−1^) for 6 days. (b) The phenotype of differentiated M2 macrophages was determined by flow cytometry and CD163 served as an M2 marker. (c,d) M2 macrophages were treated for 24 h with increasing doses of IL‐1α (c) or IL‐1ß (d) either alone or in combination with P2Y_11_ receptor agonist (ATPγS). The antagonist NF340 (10 μM) was used to confirm that agonist‐mediated responses were specific to P2Y_11_ receptor stimulation. IL‐6 and IL‐8 were measured in cell culture supernatants. Data are means ± SEM from five independent donors. *P ≤ .05, significantly different as indicated; one‐way ANOVA

The colony‐stimulating factors GM‐CSF or M‐CSF promote M1 or M2 macrophage differentiation, respectively (Hamilton, 2008). P2Y_11_ receptor gene and protein expression have been shown to increase in response to M‐CSF (Gruenbacher et al., 2019; Layhadi & Fountain, 2019), along with the scavenger receptor CD163 (Gruenbacher et al., 2019), which is an M‐CSF target gene (Buechler et al., 2000). To test the physiological relevance of our observations in the recombinant cell line, we used P2Y_11_ receptor‐expressing M2 macrophages differentiated from primary human blood monocytes in the presence of M‐CSF (50 ng·ml^−1^) (Figure 4a). In addition to CD14, which confirms their monocytic origin, these macrophages expressed CD163, indicative of M2 differentiation (Buechler et al., 2000; Mommert et al., 2020) (Figures 4b and S3A). Moreover, M2 macrophages expressed IL‐1R1 and TLR4 as well as P2X1 receptors, which are upregulated during monocyte differentiation (Wong et al., 2011) and have been implicated in anti‐inflammatory responses by controlling reactive oxygen species (ROS) production (Lecut et al., 2012).

At 200 pg·ml^−1^, both IL‐1α and IL‐1ß induced IL‐8 production in M2 macrophages (Figure S3B). Similar to the results obtained with ectopic P2Y_11_ receptors (Figure 3c,d), stimulation of native P2Y_11_ receptors on M2 macrophages enhanced IL‐8 production induced by either IL‐1α or IL‐1ß and, importantly, the synergistic response could be inhibited by NF340 (10 μM) (Figure S3B). At higher doses of IL‐1 (≥1 ng·ml^−1^), M2 macrophages produced IL‐6 in addition to IL‐8 (Figure 4c,d). Stimulation with ATPγS (10 μM) enhanced both IL‐6 and IL‐8 secretion, and NF340 effectively prevented the ATPγS effect. At IL‐1 concentrations ≥ 2 ng·ml^−1^), ATPγS no longer had a synergistic effect (Figure S4).

### 3.5 Activation of P2Y_11_ receptors promotes shedding of TNF receptors from the surface of M2 macrophages in a cyclic AMP‐ and TACE/ADAM17‐dependent manner: Synergistic enhancement by IL‐1α and IL‐1ß

**FIGURE 5:**
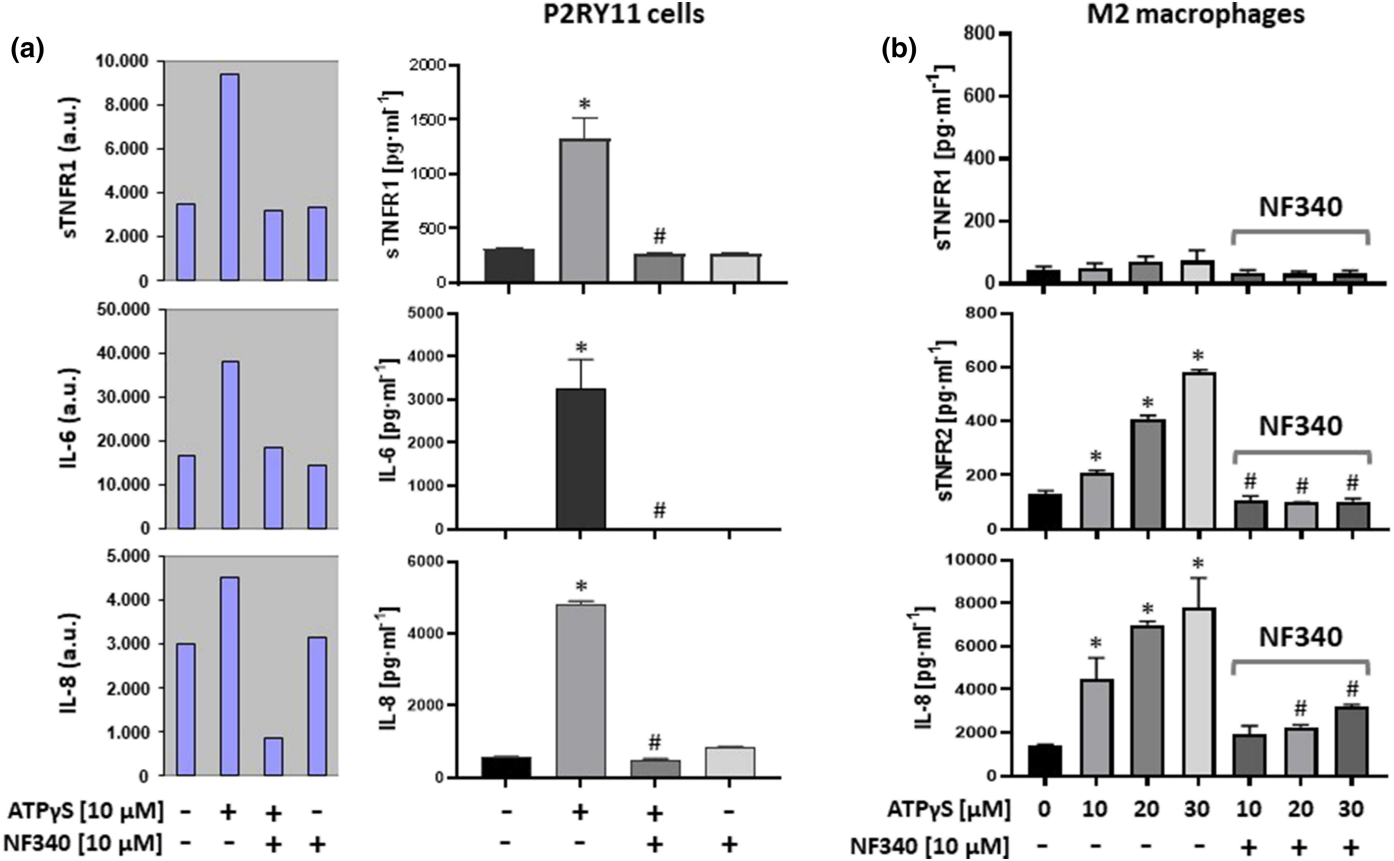
Activation of P2Y_11_ receptors stimulates the release of soluble TNF receptors. (a) P2RY11‐transfected cells were cultured for 24 h with or without P2Y_11_ receptor agonist (ATPγS). To confirm that agonist‐mediated cytokine secretion was specific to stimulation of P2Y_11_ receptors, the cells were also stimulated with agonist in the presence of the specific P2Y_11_ receptor antagonist NF340. Cell culture supernatants were subjected to a human cytokine antibody array. The G‐Series arrays are semiquantitative featuring fluorescent signal detection (a.u., arbitrary units) (left panel). sTNFR1 release was subsequently validated by ELISA (right panel). IL‐6 and IL‐8, which already emerged from the secretome analysis, served as an internal control. (b) M2 macrophages were treated for 24 h with the P2Y_11_ receptor agonist (ATPγS). The antagonist NF340 was used to confirm that agonist‐mediated responses were specific to stimulation of P2Y_11_ receptors. sTNFR1 and sTNFR2 as well as IL‐8 were measured in cell culture supernatants. Data are means ± SEM from five independent donors. *P ≤ .05, significantly different from control; ^#^ *P* < .05, significantly different from corresponding concentration of ATPγS only; one‐way ANOVA

Secretome profiling of ectopic P2Y_11_ receptor activation not only confirmed IL‐6 and IL‐8 but additionally identified sTNFR1 (Figure 5a). sTNFR1 secretion could be validated by ELISA (Figure 5a). In contrast, in our attempts to measure sTNFR2 in response to stimulation of P2Y_11_ receptors in the recombinant cell line (P2RY11), which is derived from an astrocytoma (glioma), sTNFR2 remained undetectable (0.00 pg·ml^−1^) even at agonist concentrations of up to 50 μM.

This was in accordance with the absence of sTNFR2 mRNA in the transcriptome analysis of these cells and also with the established view that TNFR2 expression is rather restricted to certain cell types such as myeloid cells (Wajant & Siegmund, 2019). Consistent with these findings, M2 macrophages did not secrete sTNFR1 but instead secreted low but significant amounts of sTNFR2 in addition to IL‐8 (Figure 5b).

**FIGURE 6:**
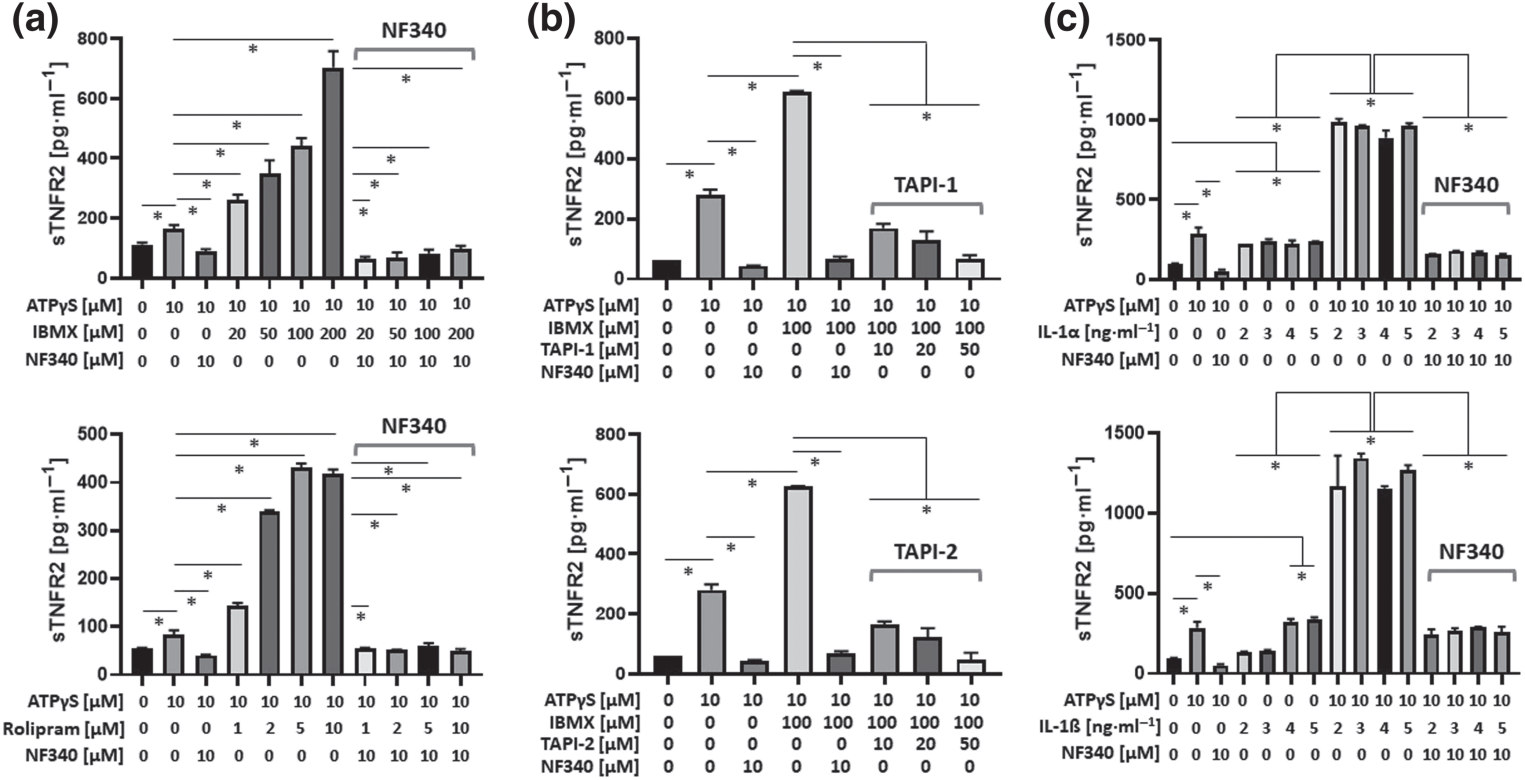
P2Y_11_ receptor‐driven release of soluble TNF receptors depends on intracellular cAMP and on TACE/ADAM17: synergistic enhancement by IL‐1α and IL‐1ß (a) M2 macrophages were treated with ATPγS in the presence or absence of increasing concentrations of the phosphodiesterase (PDE) inhibitors IBMX (nonselective) or rolipram (PDE4‐selective). The antagonist NF340 was used to confirm that agonist‐mediated responses were specific to stimulation of P2Y_11_ receptors. sTNFR2 was measured in cell culture supernatants. (b) M2 macrophages were treated with ATPγS in the presence or absence of increasing concentrations of the TACE/ADAM17 inhibitors TAPI‐1 and TAPI‐2. The antagonist NF340 was used to confirm that agonist‐mediated responses were specific to stimulation of P2Y_11_ receptors. sTNFR2 was measured in cell culture supernatants. (c) M2 macrophages were treated for 24 h with increasing doses of IL‐1α or IL‐1ß either alone or in combination with the P2Y_11_ receptor agonist ATPγS (10 μM). The antagonist NF340 (10 μM) was used to confirm that agonist‐mediated responses were specific to stimulation of P2Y_11_ receptors. For all graphs, data shown are means ± SEM from five independent donors. *P ≤ .05, significantly different as indicated; one‐way ANOVA

In addition to G_q_ coupling, P2Y_11_ receptors can also activate AC by coupling to G_s_ (Figure S2B), thus raising the intracellular levels of cAMP, which has potent anti‐inflammatory activity (Gerlo et al., 2011). Intracellular cAMP levels are lowered by phosphodiesterases (PDEs), enzymes that catalyse the degradation of cAMP to 5′AMP. To examine a potential role of cAMP in P2Y_11_ receptor‐mediated suppression of inflammation, we used IBMX, a competitive nonselective PDE inhibitor, which raises intracellular cAMP. Addition of IBMX has also been used to detect basal GPCR activity (Mathiesen et al., 2013). Although IBMX alone (20 to 200 μM) failed to stimulate release of sTNFR2 from M2 macrophages, NF340 (10 μM) displayed modest inhibitory activity in the presence of IBMX (Figure S5), indicative of weak basal activity. In addition, IBMX (20 μM to 200 μM) strongly enhanced the ATPγS‐induced release of sTNFR2 in a dose‐dependent manner, and importantly, this effect was completely prevented by NF340 (10 μM) (Figure 6a).

Since PDE4 plays a critical role in macrophage differentiation and activation (Hertz et al., 2009), we next tested the effects of PDE4 inhibition. Very much comparable to IBMX, the PDE4‐selective inhibitor rolipram (1 to 10 μM) similarly increased P2Y_11_ receptor‐driven sTNFR2 release and NF340 (10 μM) again prevented the response (Figure 6a).

ADAM17, a member of the disintegrin and metalloproteinase (ADAM) family that was initially described as tumour necrosis factor converting enzyme (TACE), can proteolytically cleave/shed membrane‐bound TNFRs causing the release of soluble TNFR ectodomains (Wajant & Siegmund, 2019). Inhibition of ADAM‐17 by both TAPI‐1 and TAPI‐2 (10 to 50 μM) prevented the release of sTNFR2 from M2 macrophages (Figure 6b). IL‐1α and IL‐1ß (2 to 5 ng·ml^−1^) also induced sTNFR2 release; however, a plateau was reached quickly (Figure 6c). In contrast, concurrent stimulation of P2Y_11_ and IL‐1 receptors resulted in a synergistic enhancement of sTNFR2 release, which could be inhibited by NF340 (10 μM), indicating that activation of P2Y_11_ receptor supported the IL‐1 receptor‐driven release of sTNFR2.

### 3.6 P2Y_11_ receptors control TLR4 signalling in M2 macrophages

TLR4, the receptor for LPS, shares the signalling pathway with IL‐1 receptors (Dunne & O'Neill, 2003) by engaging the same downstream signalling components (Figure S1). M2 macrophages expressed substantial levels of TLR4 (Figure 4b) and were highly responsive to LPS (Figure S6). Very low concentrations of LPS (10 pg·ml^−1^) already induced large amounts of IL‐8. In contrast to P2Y_11_ receptor agonists, which only stimulate IL‐8 production, TLR4 activation also increased IL‐6, TNF‐α, and IL‐10 secretion in M2 macrophages (Figure S6) (Rossol et al., 2011).

**FIGURE 7:**
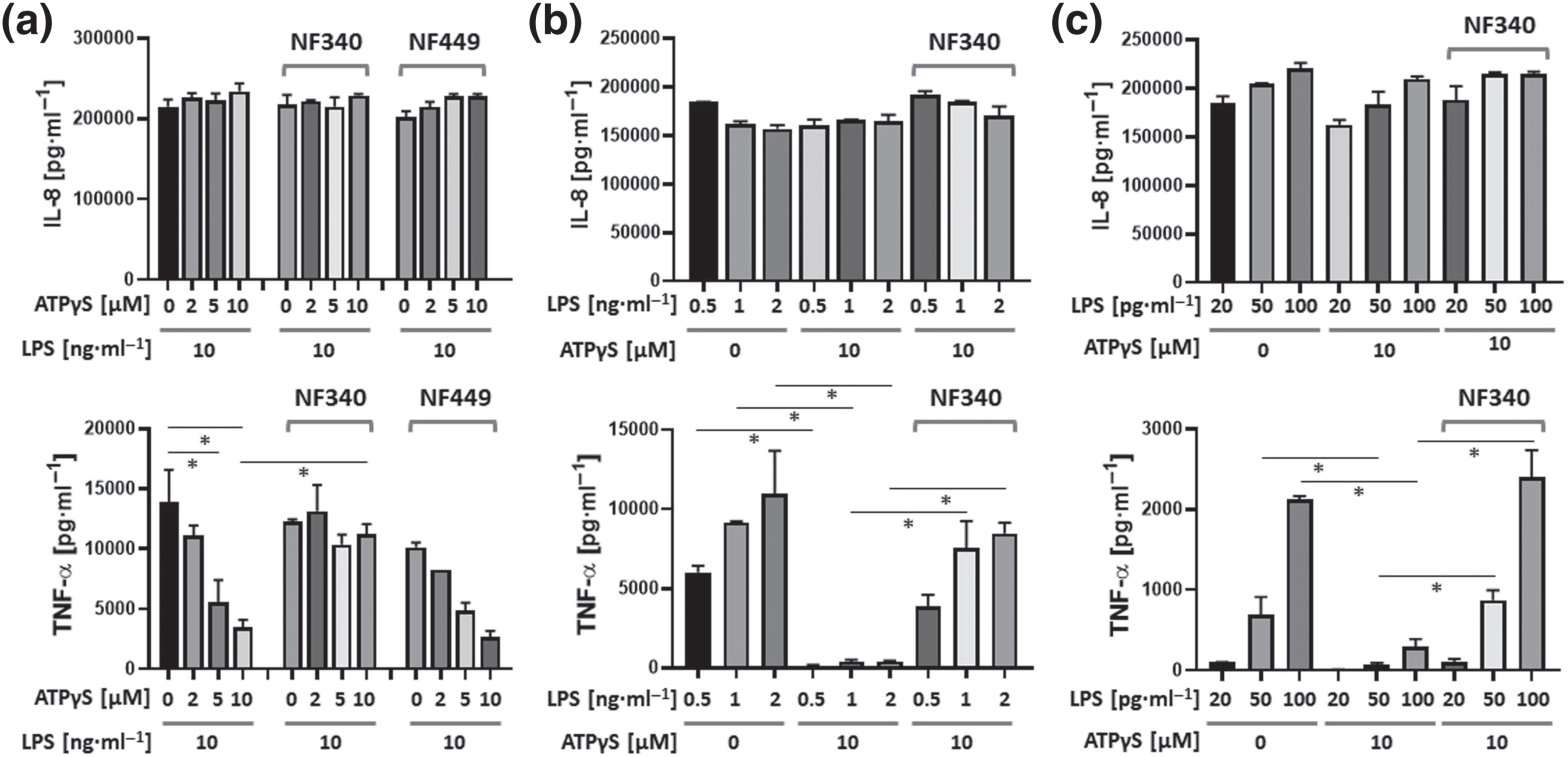
Activation of P2Y_11_ receptors inhibits LPS‐induced TNF‐α production in M2 macrophages. (a) M2 macrophages were treated with a constant dose of lipopolysaccharide (LPS) either alone or in the presence of graded doses of ATPγS. The antagonist NF340 was used to confirm that agonist‐mediated responses were specific to stimulation of P2Y_11_ receptors. NF449 was used to examine a potential role of P2X1 receptors. (b,c) M2 macrophages were treated with graded doses of LPS in the presence or absence of a constant dose of ATPγS. IL‐8 and TNF‐α were measured in cell culture supernatants. Data are means ± SEM from six independent donors. *P ≤ .05, significantly different as indicated; one‐way ANOVA

While ATPγS (2 to 10 μM) had no effect on IL‐8 production induced by LPS (10 ng·ml^−1^) (Figure 7a–c), LPS‐induced TNF‐α secretion was effectively suppressed by ATPγS in a dose‐dependent manner (Figure 7a). Importantly, TNF‐α secretion was restored by the P2Y_11_ receptor‐specific antagonist NF340 (10 μM) but not by the P2X1 receptor‐specific antagonist NF449 (10 μM) (Figure 7a). At lower concentrations of LPS (0.5 to 2.0 ng·ml^−1^), ATPγS (10 μM) inhibited TNF‐α secretion almost completely and NF340 (10 μM) substantially restored it (Figure 7b). The inhibitory effect of P2Y_11_ receptor activation on relatively strong LPS responses does not exclude the possibility that P2Y_11_ receptors might support TLR4 responses, when LPS concentrations are low. However, even at very low concentrations of LPS (20 to 100 pg·ml^−1^), TNF‐α secretion was inhibited by ATPγS (10 μM) and effectively restored by NF340 (10 μM) (Figure 7c). In contrast, LPS‐induced IL‐6, IL‐8, IL‐10, and CCL2 were not significantly regulated by activation of P2Y_11_ receptors (Figures 7a–c and S7A).

Consistent with earlier reports (Bailly et al., 1990), we found that IBMX at the same concentrations that enhanced P2Y_11_ receptor‐mediated sTNFR2 release (20–200 μM) (Figure 6a) also inhibited LPS‐induced TNF‐α secretion (Figure S7B). Altogether, these data suggested that both P2Y_11_ receptor‐driven release of sTNFR2 and P2Y_11_ receptor‐mediated suppression of TNF‐α secretion, depend on intracellular cAMP.

## 4 DISCUSSION

Although the P2RY11 gene was cloned more than two decades ago (Communi et al., 1997), many aspects of P2Y_11_ receptor signalling and function have remained unclear. This is due to the lack of a rodent knockout model and the limited availability of specific tools. The lack of specific P2Y_11_ receptor antagonists has often impeded the interpretation of data, and in fact, a number of previous reports did not fulfil the functionality criteria applied by Dreisig and Kornum in their critical P2Y_11_ receptor review (Dreisig & Kornum, 2016).

We have previously shown that P2Y_11_ receptor expression is upregulated during human M2 macrophage differentiation (Gruenbacher et al., 2019), suggesting that expression of these receptors contributes to the anti‐inflammatory function of this macrophage subset. To extend these studies, we have now examined ectopic P2Y_11_ receptors in a recombinant cell system and native P2Y_11_ receptors in human monocyte‐derived M2 macrophages. We performed the first transcriptome and secretome profiling of P2Y_11_ activation and used the suramin analogue NF340 as well as P2RY11‐knockout cells to confirm that agonist‐mediated responses were specific to stimulation of P2Y_11_ receptors.

The transcriptomic approach revealed crosstalk between P2Y_11_ and the IL‐1 receptor /TLR4 pathways, which are known to share downstream signalling components (Dunne & O'Neill, 2003). While IL‐1 receptor signalling was enhanced, TLR4 responses appeared to be suppressed. We have recently shown that P2Y_11_ receptor‐driven IL‐8 secretion requires IKK and ERK as critical signalling components, both in the ectopic expression system and in primary human M2 macrophages (Gruenbacher et al., 2019). Our current observation that P2Y_11_ receptors engage the IL‐1 receptor signalling is in accordance with our previous findings, because IL‐1 receptor responses likewise depend on IKK and ERK (Figure S1) (Weber et al., 2010).

The secretomic approach confirmed findings of the transcriptional network analysis such as the P2Y_11_ receptor‐induced secretion of IL‐6 and IL‐8, which are pleiotropic cytokines but also the hallmarks of the senescence‐associated secretory phenotype (Coppe et al., 2010). Our secretome analysis additionally identified soluble TNF receptors as novel targets of activated P2Y_11_ receptors. The synergistic enhancement of P2Y_11_ receptor‐induced sTNFR2 release from M2 macrophages by IL‐1α and IL‐1ß together with the transcriptome and secretome profiling data suggested that anti‐inflammatory P2Y_11_ receptor signalling targets IL‐1 receptors to induce shedding of TNF receptors via ADAM17. In contrast, the highly effective inhibition of LPS‐induced TNF‐α secretion upon activation of P2Y_11_ receptors indicated that anti‐inflammatory P2Y_11_ receptor signalling also targets TLR4 to prevent TNF‐α secretion. Collectively, these observations establish P2Y_11_ receptors as potent sentinels of TNF‐α induced inflammation that monitors extracellular ATP, accumulated during cell stress and tissue damage, and promptly promotes anti‐inflammatory macrophage reprogramming.

The P2Y_11_ receptor is an unconventional member of the P2Y family of GPCRs, as it can couple to G_q_ and to G_s_ proteins (Dreisig & Kornum, 2016; Kennedy, 2017). Our findings indicate that P2Y_11_ receptors couple to G_q_ to induce IL‐6 and IL‐8 expression. However, P2Y_11_ receptor coupling to G_s_ resulting in AC activation and accumulation of cAMP was required to elicit anti‐inflammatory signalling from P2Y_11_ receptors. Both P2Y_11_ receptor‐induced sTNFR2 release and P2Y_11_ receptor‐mediated suppression of LPS‐induced TNF‐α secretion from human M2 macrophages depended on intracellular cAMP, which is a well‐known second messenger with strong anti‐inflammatory potential (Gerlo et al., 2011).

P2Y_11_ receptors may also be involved in endotoxin tolerance, which is a negative form of trained immunity (Vergadi et al., 2018) with great clinical relevance in conditions of sepsis (Biswas & Lopez‐Collazo, 2009). Endotoxin tolerance refers to a prolonged state of hyporesponsiveness after strong TLR4 activation by LPS. Stable downregulation of TNF‐α is observed in many models of tolerisation and is thus considered a hallmark of endotoxin tolerance (Biswas & Lopez‐Collazo, 2009). LPS‐mediated TLR4 activation is known to cause the release of ATP from macrophages (Piccini et al., 2008), which enhances inflammation via inflammasome‐dependent generation and secretion of IL‐1ß and IL‐18. In accordance with our current findings, however, extracellular ATP may also induce P2Y_11_ receptor‐mediated suppression of TNF‐α and thus establish the sepsis‐associated hyporesponsive state.

Although the signalling mechanisms involved in SARS‐CoV‐2‐mediated activation of inflammatory macrophage responses are still unclear, IL‐1 receptor/TLR pathways are considered to play a critical role (Merad & Martin, 2020; Netea et al., 2020). Various TLRs can be activated by members of the SARS‐CoV family of coronaviruses. In addition, transcriptional upregulation of MyD88‐dependent IL‐1/TLR pathways and cytokine responses has been demonstrated in COVID‐19 patients (Ong et al., 2020). Moreover, TNF‐α, a major downstream effector of TLR signalling, plays a critical role in hyperinflammation associated with COVID‐19 and is therefore considered an attractive therapeutic target (Catanzaro et al., 2020). Our current findings that P2Y_11_ receptors engage the MyD88‐dependent IL‐1 receptor/TLR signalling pathways to neutralise TNF‐α and to suppress further TNF‐α secretion raises the possibility that P2Y_11_ receptor‐expressing M2 macrophages support milder courses of the disease. Targeting P2Y_11_ receptors with agonists to elicit anti‐inflammatory P2Y_11_ receptor signalling in M2 macrophages may thus be desirable to prevent deleterious inflammation in severe forms of COVID‐19, characterised by excessive M1 macrophage activation (Merad & Martin, 2020).

In conclusion, uncontrolled inflammation leads to increased morbidity and mortality in patients with infectious diseases. The G protein‐coupled ATP receptor P2Y_11_ is considered a sensor of cell stress promoting cytoprotective responses. However, P2Y_11_ receptors have been difficult to study due to the lack of rodent knockout models and the limited availability of specific tools. By performing the first transcriptome and secretome profiling of P2Y_11_ receptor activation, we were able to demonstrate crosstalk between P2Y_11_ receptor and IL‐1 receptor /TLR signalling pathways. Activation of P2Y_11_ receptors thus promoted the release of soluble TNF receptors and suppressed TNF‐α secretion from human M2 macrophages. The current findings improve our understanding of P2Y_11_ receptor signalling in anti‐inflammatory macrophages and have implications for the treatment of hyperinflammation such as that associated with COVID‐19.

## AUTHOR CONTRIBUTIONS

G.G. and M.T. designed and supervised the study. H.G., G.D., and D.K. performed monocyte isolation, differentiation, and stimulation as well as propagation of cell lines. A.R. carried out cytokine measurements. G.G. operated the Canto II flow cytometer. G.G., M.T., and D.K. analysed and interpreted data. M.T., G.G., and D.K. prepared graphs, wrote, and revised the manuscript. All authors reviewed, revised, and approved the manuscript for publication.

## CONFLICT OF INTEREST

The authors declare no conflicts of interest.

## DECLARATION OF TRANSPARENCY AND SCIENTIFIC RIGOUR

This Declaration acknowledges that this paper adheres to the principles for transparent reporting and scientific rigour of preclinical research as stated in the *BJP* guidelines for Design & Analysis, Immunoblotting and Immunochemistry, and Animal Experimentation, and as recommended by funding agencies, publishers, and other organisations engaged with supporting research.

## FUNDING INFORMATION

This work was supported by the Austrian Science Fund (P28923‐B28 and P33640‐B to MT).

## Supporting information


**FIGURE S1** P2Y_11_ governs IL‐1R and TLR4, which share downstream signaling components. IL‐1R/TLR4 activation leads to the association of MyD88 (myleoid differentiation primary‐response protein 88). As a result, interleukin‐1 receptor‐associated kinase 1 (IRAK1) and IRAK4 are recruited to the receptor. Following IRAK1 phosphorylation by IRAK4, TNF receptor‐associated factor 6 (TRAF6) interacts with IRAK1. This leads to the dissociation and relocation of IRAK1, IRAK4, and TRAF6 to the plasma membrane, where interaction with the complex of TGF‐β‐ activated kinase 1 (TAK1) and TAK1‐binding proteins leads to the relocation and association of TAK1 to the IκB kinase (IKK) complex. Interaction with IKK leads to IκB degradation and NF‐κB transcription factor activity. TAK1 is also required for MAPK/ERK/AP‐1 activation. Earlier data and current findings collectively suggest that P2Y_11_ signaling via G_q_ promotes IL‐1R signaling. Raising intracellular cyclic AMP levels, for instance, via phosphodiesterase (PDE) inhibition inhibits NF‐κB, enhances the P2Y_11_/IL‐1R‐driven release of soluble tumor necrosis factor (TNF) receptors and blocks LPS‐induced TNF‐α secretion.
Figure S2 (A) Dose–response of ectopic P2Y_11_ activation. P2Y11R cells were stimulated for 24 h with agonist at increasing concentrations in the presence or absence of the antagonist NF340 (10 μM). IL‐6 and IL‐8 were measured in cell culture supernatants. Data are means ± SEM from four independent cell populations (n = 5). (B) Schematic representation of P2Y11 and its coupling to G proteins. (C) P2Y_11_R cells were stimulated for 24 h with agonist in the presence or absence of increasing concentrations of recombinant IL‐1RA (n = 5). For statistical analyses, One‐Way ANOVA was calculated. *p ≤ 0.05;
FIGURE S3 (A) M2 macrophage differentiation. CD163 and P2Y_11_ have been stained on freshly isolated monocytes (d0) and on macrophages cultured with M‐CSF (50 ng·ml^−1^) for 6 days (d6). Upregulation of CD163 and P2Y_11_ during M‐CSF driven M2 macrophage differentiation has been shown previously (Gruenbacher et al., 2019). CD163 is an M‐CSF target gene (Buechler et al., 2000) and thus serves as a marker of M2 differentiation. (B) Native P2Y_11_ enhances IL‐1R signaling. Primary human monocytes were obtained from peripheral blood mononuclear cells (PBMCs) by magnetic‐activated cell sorting (MACS) using CD14 microbeads. M2 macrophages were differentiated by culturing the isolated monocytes with M‐CSF (50 ng·ml^−1^) for 6 days. M2 macrophages were treated for 24 h with increasing doses of IL‐1α (A) or IL‐1ß (B) either alone or in combination with P2Y_11_ agonist (ATPγS). NF340 (10 μM) was used to confirm that agonist‐mediated responses were specific to P2Y11 stimulation. IL‐8 was measured in cell culture supernatants (n = 5). For statistical analyses, One‐Way ANOVA was calculated. *p ≤ 0.05;
FIGURE S4 P2Y_11_ does not enhance IL‐1R signaling, when IL‐1 concentrations are high. (A‐B) M2 macrophages were treated for 24 h with increasing doses of IL‐1α (A) or IL‐1ß (B) either alone or in combination with P2Y_11_ agonist (ATPγS). NF340 (10 μM) was used to confirm that agonist‐mediated responses were specific to P2Y_11_ stimulation. IL‐8 was measured in cell culture supernatants (n = 5). For statistical analyses, One‐Way ANOVA was calculated.
FIGURE S5 Phosphodiesterase inhibition is not sufficient to induce the release of soluble TNF receptors. M2 macrophages were treated with increasing concentrations of the non‐selective phosphodiesterase inhibitor IBMX in the presence or absence of the P2Y_11_ inhibitor NF340. sTNFR2 was measured in cell culture supernatants (n = 5). For statistical analyses, One‐Way ANOVA was calculated. *p < 0.05;
FIGURE S6 M2 macrophages are highly LPS‐responsive. M2 macrophages were treated with graded doses of lipopolysaccharide (LPS). IL‐8, IL‐6, TNF‐α and IL‐10 were measured in cell culture supernatants. LPS‐induced production of IL‐6, IL‐8, TNF‐α and IL‐10 in macrophages is well established (Rossol et al., 2011).
FIGURE S7 P2Y_11_ abrogates LPS‐induced TNF‐α production in M2 macrophages but has little or no effect on CCL2, IL‐6 and IL‐10. (A) M2 macrophages were treated with a constant dose of lipopolysaccharide (LPS) either alone or in the presence of graded doses of ATPγS. NF340 was used to confirm that agonist‐mediated responses were specific to P2Y_11_ stimulation. NF449 was used to examine a potential role of P2X1. (B) IBMX abrogates LPS‐induced TNF‐α production in M2 macrophages. M2 macrophages were treated with LPS in the presence or absence of graded doses of IBMX. TNF‐α was measured in cell culture supernatants. The inhibitory effect of IBMX on LPS‐induced TNF‐α production is well established (Bailly, Ferrua, Fay, & Gougerot‐Pocidalo, 1990).

## Data Availability

The data that support the findings of this study are available from the corresponding author upon reasonable request. Additional transcriptome and secretome profiling data are also available on request from the corresponding author (M.T.). Some data may not be made available because of privacy or ethical restrictions.
