# Supplementary material for: The human G protein‐coupled ATP receptor P2Y11 is a target for anti‐inflammatory strategies
Source: Br J Pharmacol. 2021 Feb 19;178(7):1541–55. doi: 10.1111/bph.15379 (PMC9328440; doi:10.1111/bph.15379)
Supplement: Supplementary file 1 — FIGURE S1 P2Y11 governs IL‐1R and TLR4, which share downstream signaling components. IL‐1R/TLR4 activation leads to the association of MyD88 (myleoid differentiation primary‐response protein 88). As a result, interleukin‐1 receptor‐associated kinase 1 (IRAK1) and IRAK4 are recruited to the receptor. Following IRAK1 phosphorylation by IRAK4, TNF receptor‐associated factor 6 (TRAF6) interacts with IRAK1. This leads to the dissociation and relocation of IRAK1, IRAK4, and TRAF6 to the plasma membrane, where interaction with the complex of TGF‐β‐ activated kinase 1 (TAK1) and TAK1‐binding proteins leads to the relocation and association of TAK1 to the IκB kinase (IKK) complex. Interaction with IKK leads to IκB degradation and NF‐κB transcription factor activity. TAK1 is also required for MAPK/ERK/AP‐1 activation. Earlier data and current findings collectively suggest that P2Y11 signaling via Gq promotes IL‐1R signaling. Raising intracellular cyclic AMP levels, for instance, via phosphodiesterase (PDE) inhibition inhibits NF‐κB, enhances the P2Y11/IL‐1R‐driven release of soluble tumor necrosis factor (TNF) receptors and blocks LPS‐induced TNF‐α secretion. Figure S2 (A) Dose–response of ectopic P2Y11 activation. P2Y11R cells were stimulated for 24 h with agonist at increasing concentrations in the presence or absence of the antagonist NF340 (10 μM). IL‐6 and IL‐8 were measured in cell culture supernatants. Data are means ± SEM from four independent cell populations (n = 5). (B) Schematic representation of P2Y11 and its coupling to G proteins. (C) P2Y11R cells were stimulated for 24 h with agonist in the presence or absence of increasing concentrations of recombinant IL‐1RA (n = 5). For statistical analyses, One‐Way ANOVA was calculated. *p ≤ 0.05; FIGURE S3 (A) M2 macrophage differentiation. CD163 and P2Y11 have been stained on freshly isolated monocytes (d0) and on macrophages cultured with M‐CSF (50 ng·ml−1) for 6 days (d6). Upregulation of CD163 and P2Y11 during M‐CSF d [file BPH-178-1541-s001.pdf]

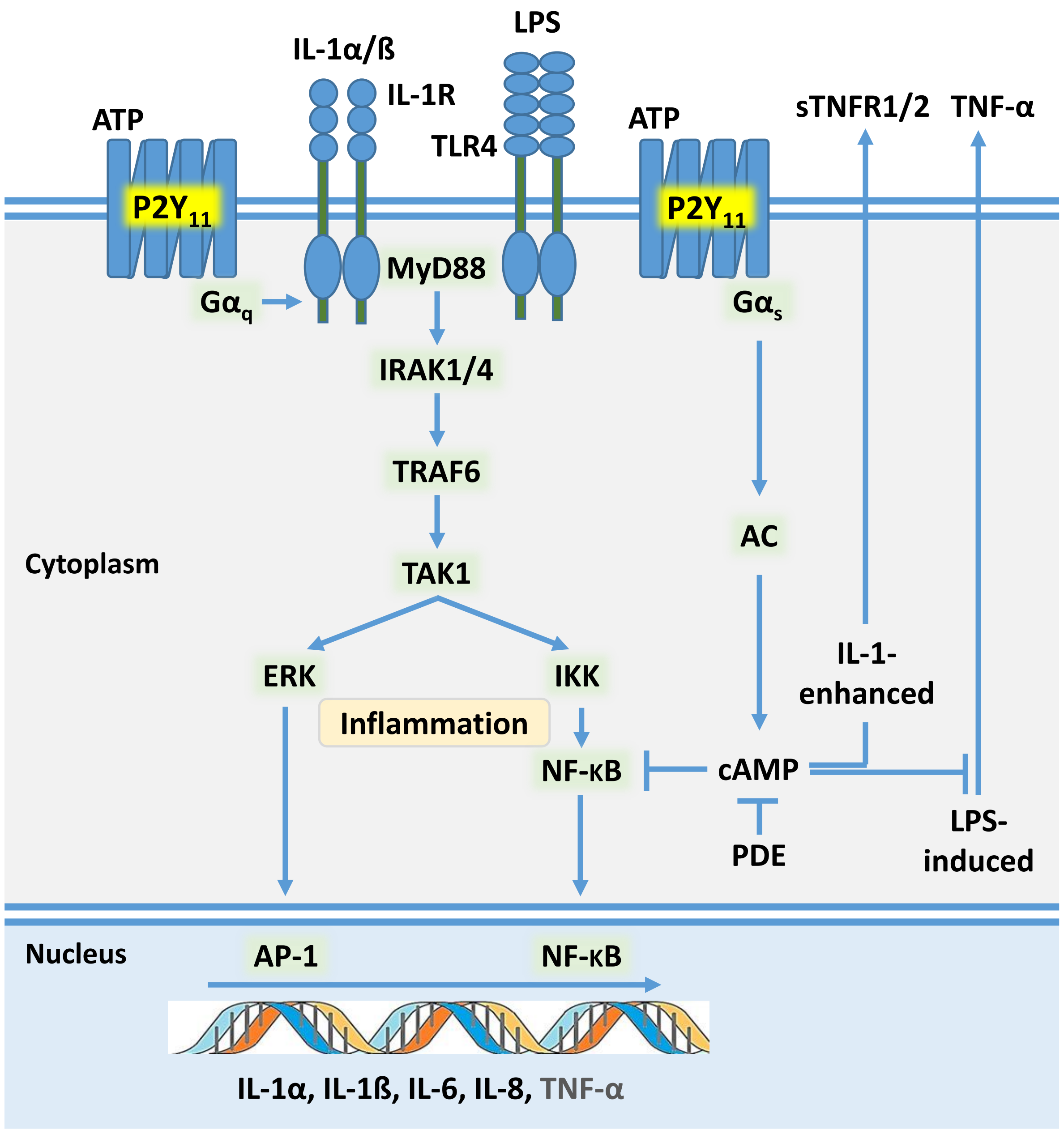

FIGURE S1 $P2Y_{11}$ governs IL-1R and TLR4, which share downstream signaling components. IL-1R/TLR4 activation leads to the association of MyD88 (myleoid differentiation primary-response protein 88). As a result, interleukin-1 receptor-associated kinase 1 (IRAK1) and IRAK4 are recruited to the receptor. Following IRAK1 phosphorylation by IRAK4, TNF receptor-associated factor 6 (TRAF6) interacts with IRAK1. This leads to the dissociation and relocation of IRAK1, IRAK4, and TRAF6 to the plasma membrane, where interaction with the complex of TGF-β-activated kinase 1 (TAK1) and TAK1-binding proteins leads to the relocation and association of TAK1 to the IκB kinase (IKK) complex. Interaction with IKK leads to IκB degradation and NF-κB transcription factor activity. TAK1 is also required for MAPK/ERK/AP-1 activation. Earlier data and current findings collectively suggest that $P2Y_{11}$ signaling via G_q promotes IL-1R signaling. Raising intracellular cyclic AMP levels, for instance, via phosphodiesterase (PDE) inhibition inhibits NF-κB, enhances the $P2Y_{11}$/IL-1R-driven release of soluble tumor necrosis factor (TNF) receptors and blocks LPS-induced TNF-α secretion.

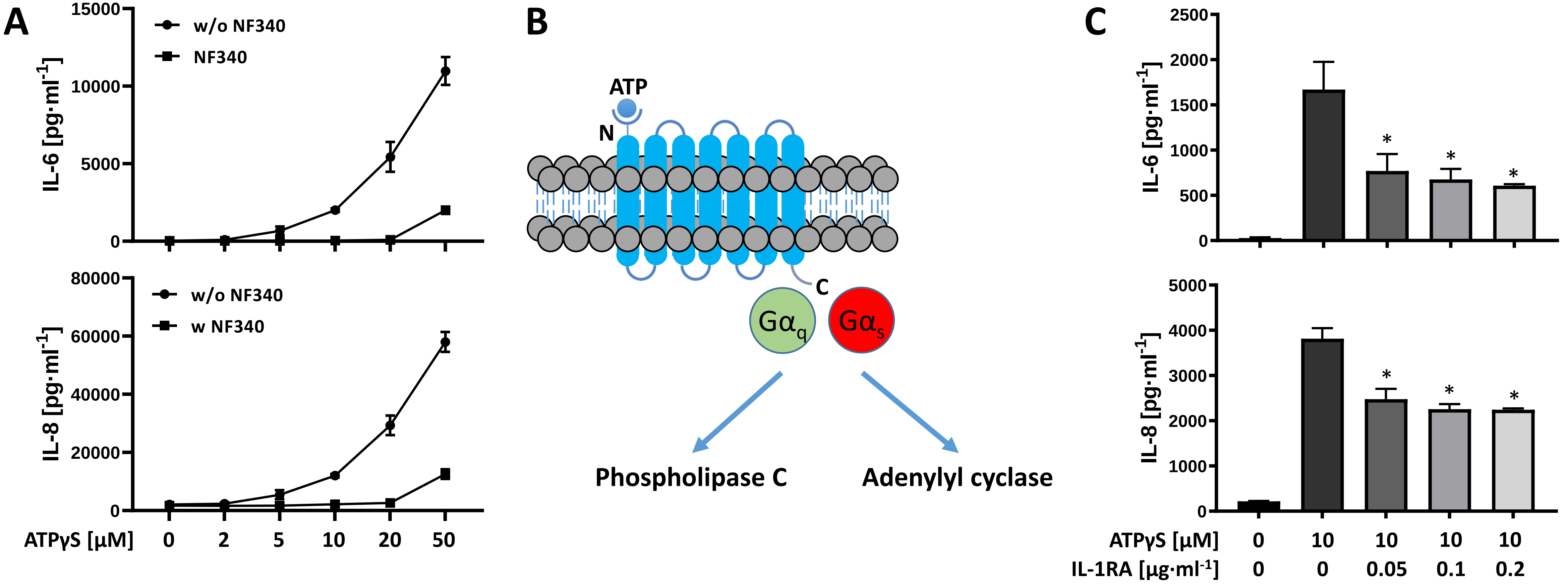

Figure S2 (A) Dose-response of ectopic $P2Y_{11}$ activation. P2Y11R cells were stimulated for 24 h with agonist at increasing concentrations in the presence or absence of the antagonist NF340 (10 µM). IL-6 and IL-8 were measured in cell culture supernatants. Data are means ± SEM from four independent cell populations (n=5). (B) Schematic representation of $P2Y_{11}$ and its coupling to G proteins. (C) P2Y11R cells were stimulated for 24 h with agonist in the presence or absence of increasing concentrations of recombinant IL-1RA (n=5). For statistical analyses, One-Way ANOVA was calculated. *p≤ 0.05;

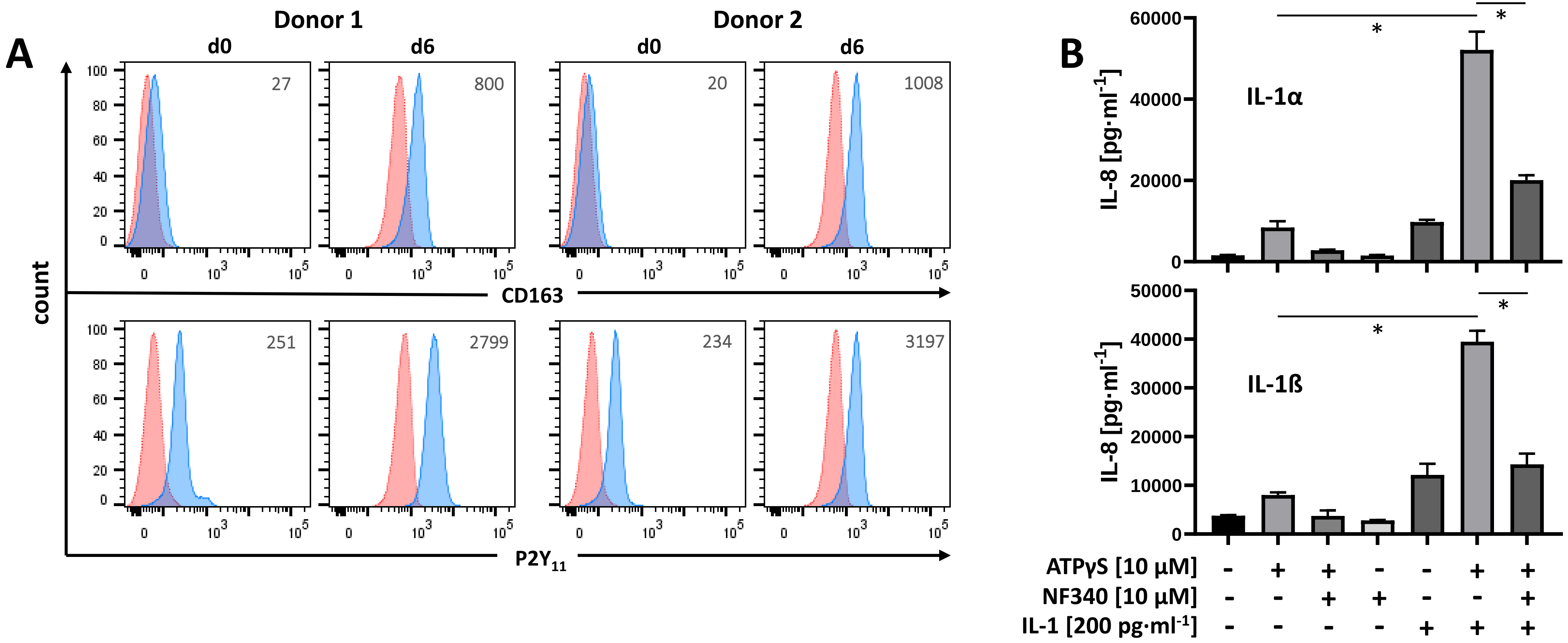

FIGURE S3 (A) M2 macrophage differentiation. CD163 and $P2Y_{11}$ have been stained on freshly isolated monocytes (d0) and on macrophages cultured with M-CSF (50 ng·ml^{-1}) for 6 days (d6). Upregulation of CD163 and $P2Y_{11}$ during M-CSF driven M2 macrophage differentiation has been shown previously (Gruenbacher et al. 2019). CD163 is an M-CSF target gene (Buechler et al. 2000) and thus serves as a marker of M2 differentiation. (B) Native $P2Y_{11}$ enhances IL-1R signaling. Primary human monocytes were obtained from peripheral blood mononuclear cells (PBMCs) by magnetic-activated cell sorting (MACS) using CD14 microbeads. M2 macrophages were differentiated by culturing the isolated monocytes with M-CSF (50 ng·ml^{-1}) for 6 days. M2 macrophages were treated for 24 h with increasing doses of IL-1α (A) or IL-1ß (B) either alone or in combination with $P2Y_{11}$ agonist (ATPγS). NF340 (10 µM) was used to confirm that agonist-mediated responses were specific to $P2Y_{11}$ stimulation. IL-8 was measured in cell culture supernatants (n=5). For statistical analyses, One-Way ANOVA was calculated. *$p \leq 0.05$;

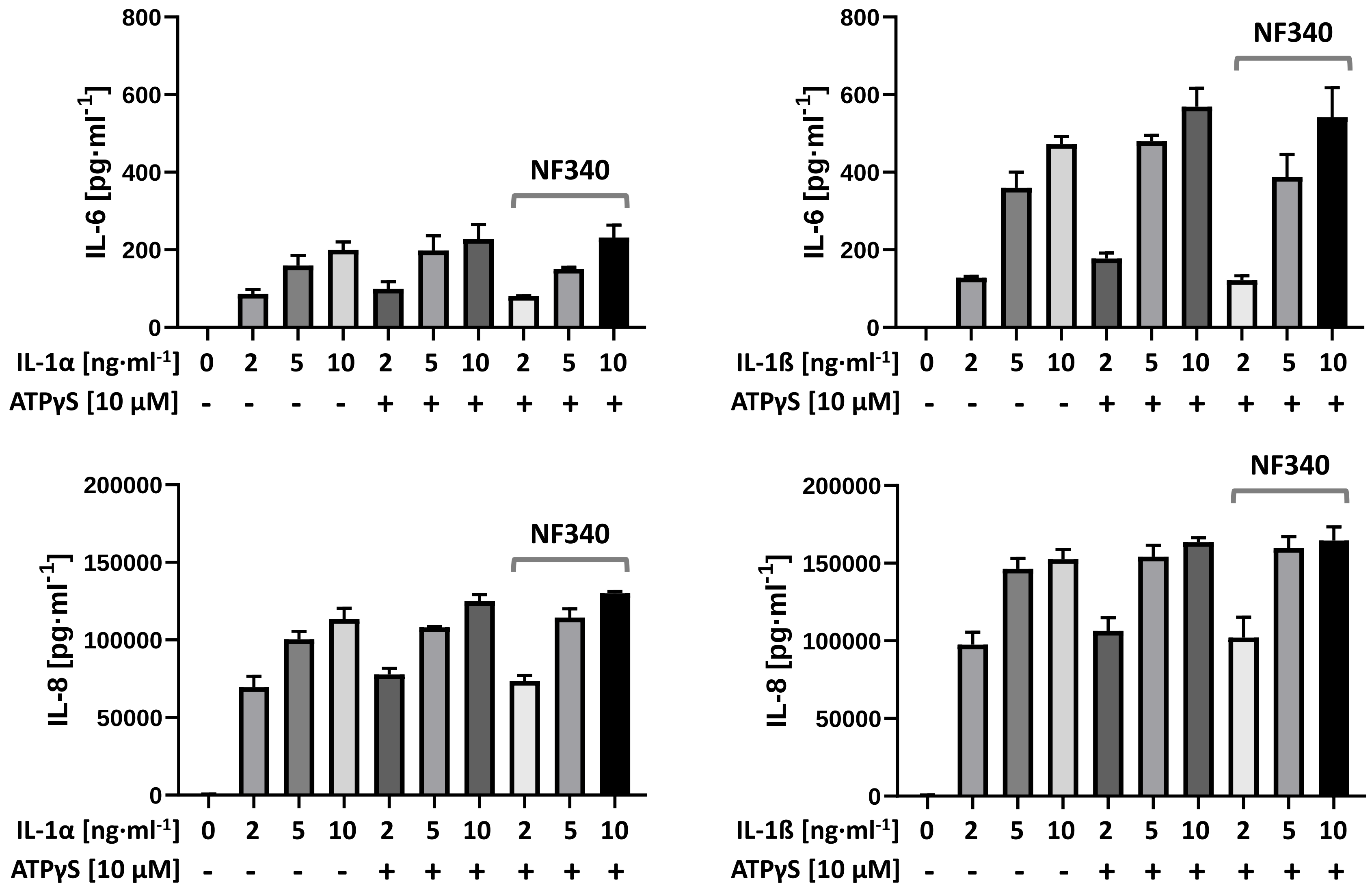

FIGURE S4 $P2Y_{11}$ does not enhance IL-1R signaling, when IL-1 concentrations are high. (A-B) M2 macrophages were treated for 24 h with increasing doses of IL-1α (A) or IL-1ß (B) either alone or in combination with $P2Y_{11}$ agonist (ATPγS). NF340 (10 µM) was used to confirm that agonist-mediated responses were specific to $P2Y_{11}$ stimulation. IL-8 was measured in cell culture supernatants (n=5). For statistical analyses, One-Way ANOVA was calculated.

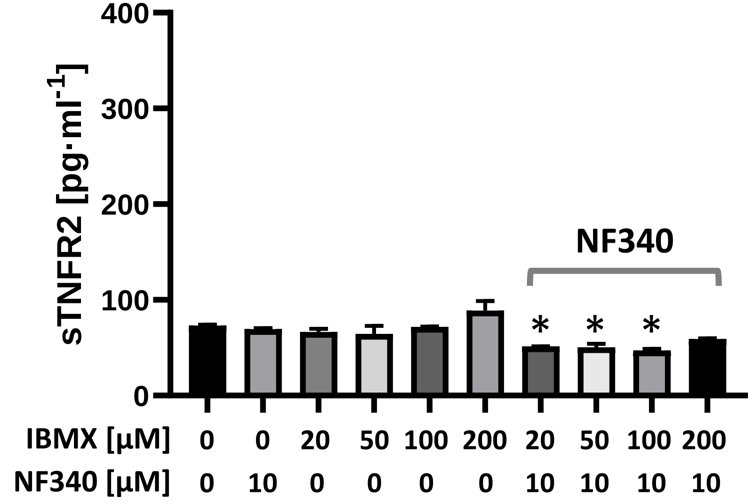

FIGURE S5 Phosphodiesterase inhibition is not sufficient to induce the release of soluble TNF receptors. M2 macrophages were treated with increasing concentrations of the non-selective phosphodiesterase inhibitor IBMX in the presence or absence of the $P2Y_{11}$ inhibitor NF340. sTNFR2 was measured in cell culture supernatants (n=5). For statistical analyses, One-Way ANOVA was calculated. *$p < 0.05$;

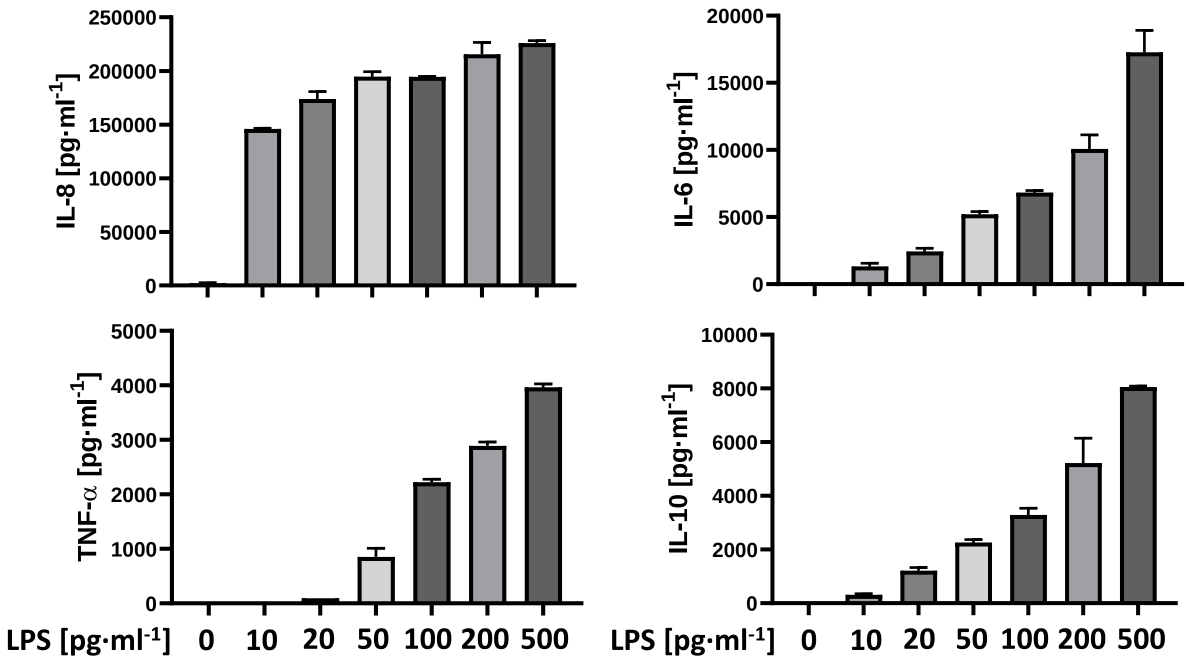

FIGURE S6 M2 macrophages are highly LPS-responsive. M2 macrophages were treated with graded doses of lipopolysaccharide (LPS). IL-8, IL-6, TNF-α and IL-10 were measured in cell culture supernatants. LPS-induced production of IL-6, IL-8, TNF-α and IL-10 in macrophages is well established (Rossol et al. 2011).

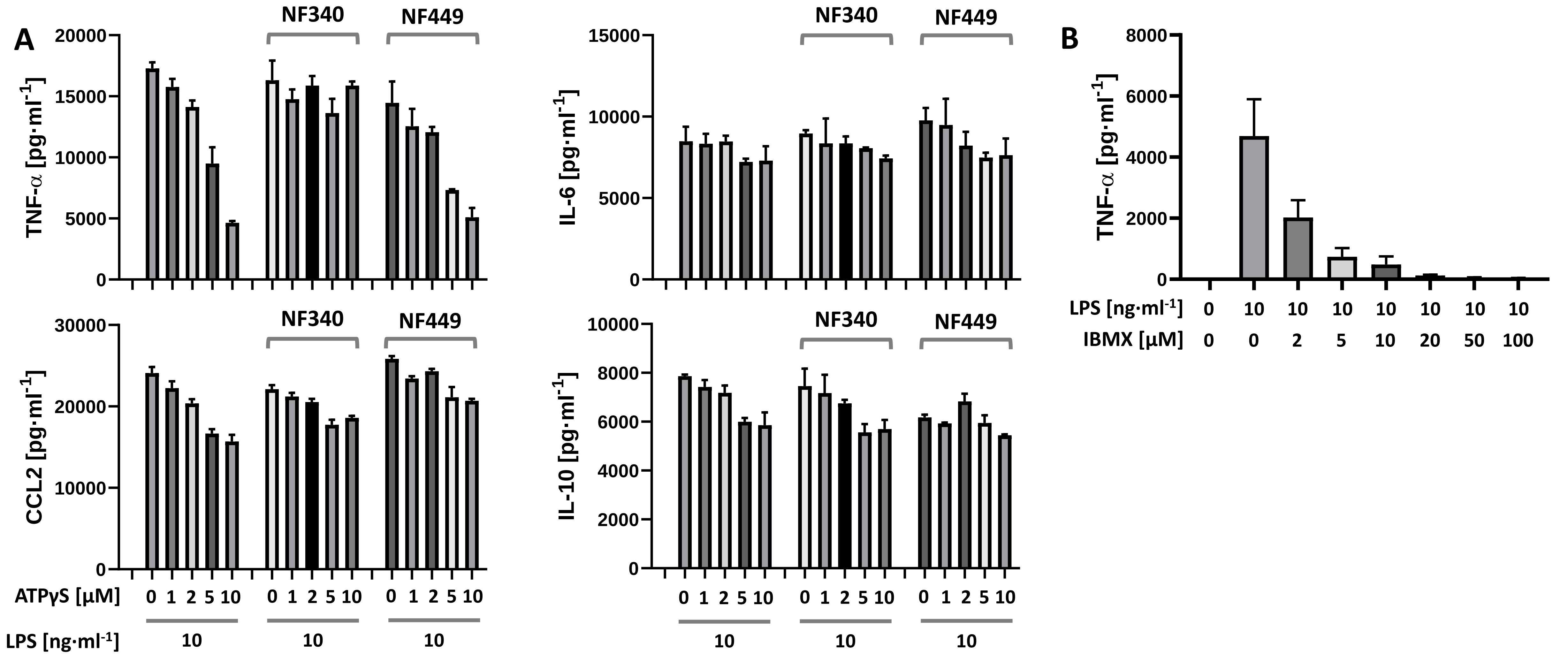

FIGURE S7 P2Y$_{11}$ abrogates LPS-induced TNF-α production in M2 macrophages but has little or no effect on CCL2, IL-6 and IL-10. (A) M2 macrophages were treated with a constant dose of lipopolysaccharide (LPS) either alone or in the presence of graded doses of ATPγS. NF340 was used to confirm that agonist-mediated responses were specific to P2Y$_{11}$ stimulation. NF449 was used to examine a potential role of P2X$_1$. (B) IBMX abrogates LPS-induced TNF-α production in M2 macrophages. M2 macrophages were treated with LPS in the presence or absence of graded doses of IBMX. TNF-α was measured in cell culture supernatants. The inhibitory effect of IBMX on LPS-induced TNF-α production is well established (Bailly et al. 1990).
